# The Chemical Composition and Antimitotic, Antioxidant, Antibacterial and Cytotoxic Properties of the Defensive Gland Extract of the Beetle, *Luprops tristis* Fabricius

**DOI:** 10.3390/molecules27217476

**Published:** 2022-11-02

**Authors:** Ovungal Sabira, Attuvalappil Ramdas Vignesh, Anthyalam Parambil Ajaykumar, Sudhir Rama Varma, Kodangattil Narayanan Jayaraj, Merin Sebastin, Kalleringal Nikhila, Annet Babu, Vazhanthodi Abdul Rasheed, Valiyaparambil Sivadasan Binitha, Zeena koldath Vasu, Madathilpadi Subrahmanian Sujith

**Affiliations:** 1 Division of Biomaterial Sciences, Department of Zoology, Sree Neelakanta Government Sanskrit College, Pattambi 679306, India; 2 Clinical Sciences Department, Centre for Medical and Bio-Allied Health Sciences Research, Ajman University, Ajman P.O. Box 346, United Arab Emirates; 3 Basic Sciences Department, Centre for Medical and Bio-Allied Health Sciences Research, Ajman University, Ajman P.O. Box 346, United Arab Emirates; 4 Department of Zoology, Sree Narayana College, Nattika, Thrissur 680566, India

**Keywords:** *Luprops tristis*, Mupli beetle, GC-HRMS analysis, defensive gland, Coleoptera

## Abstract

The unpredictable invasion of the Mupli beetle, *Luprops tristis* Fabricius (Coleoptera: Tenebrionidae), makes areas uninhabitable to humans. These beetles produce a strong-smelling, irritating secretion as a defence mechanism, which causes blisters on contact with human skin. In the current study, gas chromatography high-resolution mass spectrometry (GC-HRMS) analysis of the defensive gland extract of the Mupli beetle revealed the presence of compounds such as 2,3,dimethyl-1,4-benzoquinone, 1,3-dihydroxy-2-methylbenzene, 2,5-dimethyl hydroquinone, tetracosane, oleic acid, hexacosane, pentacosane, 7-hexadecenal and tert-hexadecanethiol. The defensive gland extracts showed considerable antibacterial activity on Gram-negative and Gram-positive bacteria in an agar diffusion assay. The chromosomal aberration analysis using root tips of *Allium cepa* L. exposed to the defensive secretion showed chromosomal aberrations such as disturbed metaphase, sticky chromosomes and chromosomal breakage. The antioxidant activity of the extract was determined using a radical scavenging (DPPH) assay. A cytotoxic assay of the defensive gland extract against Dalton’s lymphoma ascites (DLA) cell line showed anticancer properties. In the present study, the defensive gland extract of the Mupli beetle, *L. tristis*, which is generally perceived as a nuisance insect to humans, was found to have beneficial biological activities.

## 1. Introduction

Coleoptera is the largest order of insects, and Tenebrionidae is a prominent family within this order. Most species of Tenebrionidae are found in rotten wood, under logs and the bark of old trees. Both the adults and larvae feed on plant materials, decaying vegetation, etc. [1]. Based on the structure of mouth parts, male genitalia and the occurrence of a defensive gland, two broad evolutionary lineages of tenebrionid beetles are recognized; in these, tentyrioids lack defensive glands, and the tenebrionoid lineage possesses abdominal defensive glands. Many Coleopteran families possess abdominal defensive glands, but a comparative investigation was performed only in Carabidae and Tenebrionidae [2]. Comparative investigations of the compounds in defensive gland extract reveal that it has systematic value without focusing on its biological applications [3]. Gas–liquid chromatography of the chemical constituents of the defensive gland secretion of different species of Tenebrionidae indicates the presence of toluquinone, ethylquinone and benzoquinone [4]. In addition to quinones, previously unidentified 4-methyl ketones and unsaturated ketones are also identified in the defensive secretion [5].

Despite the fact that many studies on the biochemical components of the defensive gland secretions of beetles have been carried out in various countries [6], studies on Indian beetles are comparatively rare [7]. There are no available data on the biomolecules present in the defensive secretion of the members of the Coleopteran genus, *Luprops*. The experimental organism *L. tristis* is a darkling beetle, which produces an odoriferous secretion when it gets disturbed that causes skin blisters in humans. The defensive glands are invaginations of the intersegmental membrane between the seventh and eighth sternites of the beetle, which open backward and everted on pressing the abdomen. When the beetle is disturbed, this gland is ruptured by rubbing with the hind tarsus for the release of the secretion as a part of a defence mechanism against predators [8]. Biomolecules of both plant and animal origin with antimitotic, antibacterial, cytotoxic and antioxidant properties are demonstrated to have important therapeutic uses. The biologically significant metabolites in animals have attracted much attention from the scientific community. The main objective of the current study is to identify the chemical components of the defensive secretion of the Mupli beetle, *L. tristis*. In addition, we have analysed bioassays to examine the antimitotic, antibacterial, free radical scavenging and cytotoxic properties of the defensive glandular extract of the beetle, *L. tristis*.

## 2. Results

### 2.1. GC-HRMS Analysis

**Figure 1:**
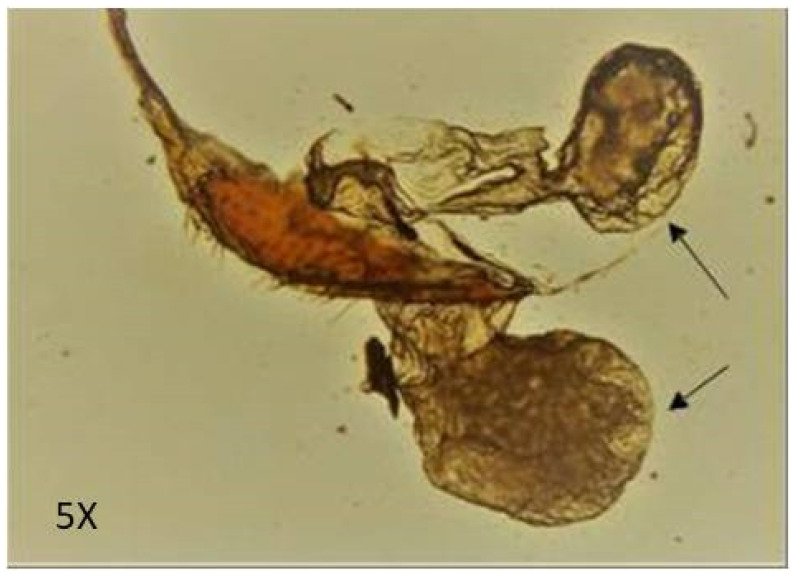
The defensive gland of the Mupli beetle, *Luprops tristis.* Arrows indicate the reservoir region of the gland.

**Figure 2:**
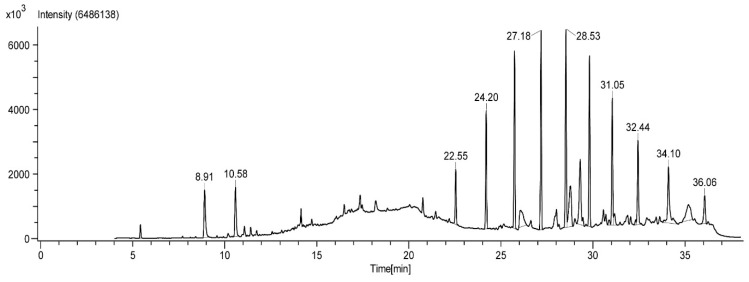
Gas chromatography high-resolution mass spectrometry (GC-HRMS) chromatogram of the defensive gland extracts of the beetle, *L. tristis*.

**Figure 3:**
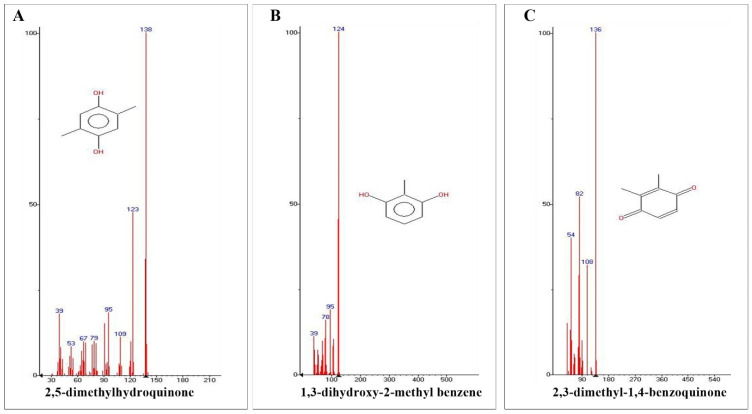
GC-HRMS analysis of identified quinone and phenolic compounds in the defensive gland extract of *L. tristis*. Data show the presence of 2,5-dimethylhydroquinone (**A**), 1,3-dihydroxy-2-methylbenzene (**B**) and 2,3-dimethyl-1,4-benzoquinone (**C**).

The adult beetle possesses two tiny defensive glands that measure about 0.8–0.9 mm in size (Figure 1). The chemical composition of the defensive gland secretion of *L. tristis* was analysed using the gas chromatography high-resolution mass spectrometry (GC-HRMS) technique. The GC-HRMS data are presented in Figure 2. The results show that the defensive secretion of *L. tristis* consists of 2,3-dimethyl-1,4-benzoquinone, 1,3-dihydroxy-2-methylbenzene, 2,5-dimethylhydroquinone, tetracosane, oleic acid, hexacosane, pentacosane, 7-hexadecenal and tert-hexadecanethiol (Figure 3 and Table 1).

### 2.2. Antimitotic Activity

**Figure 4:**
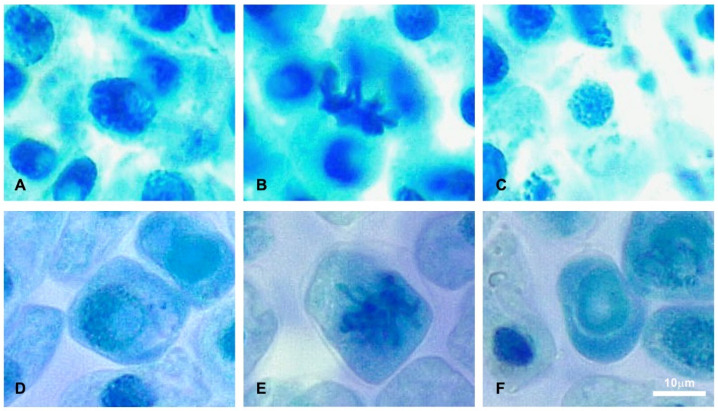
The effect of the defensive gland extract on dividing *A. cepa* cells. (**A**) Normal prophase, (**B**) normal metaphase, (**C**) normal nuclei, (**D**) disintegrated prophase, (**E**) abnormal metaphase and (**F**) disintegrated nuclei.

Various chromosomal aberrations were observed in the mitotic chromosomes when different concentrations of defensive secretion of *L. tristis* were applied to growing root meristem cells of *A. cepa* (Figure 4, Table 2). The frequency of chromosomal aberrations gradually increased in the experimental group along with an increasing concentration of the defensive gland extract (Table 2). The defensive extract hindered the normal mitotic cell division process. The aberrations are in the form of disturbed metaphase, sticky chromosomes and chromosomal breakage. However, the control group showed normal mitotic cell division without any aberrations. 

### 2.3. Antioxidant Activity

The DPPH, ABTS radical scavenging, peroxide scavenging and ferric reducing antioxidant power assays were used to investigate the antiradical scavenging efficiency of the defensive secretion of *L. tristis*. The half-maximal inhibitory concentration (IC50) for the defensive gland extract was 108.3 ± 2.1 µg/mL, 95.6 ± 1.5 µg/mL, 69.4 ± 3.5 µg/mL and 34.1 ± 1.0 µg/mL for the DPPH, ABTS radical scavenging, peroxide scavenging and iron reducing antioxidant capacity assays, respectively (Table 3). 

### 2.4. Antibacterial Activity

**Figure 5:**
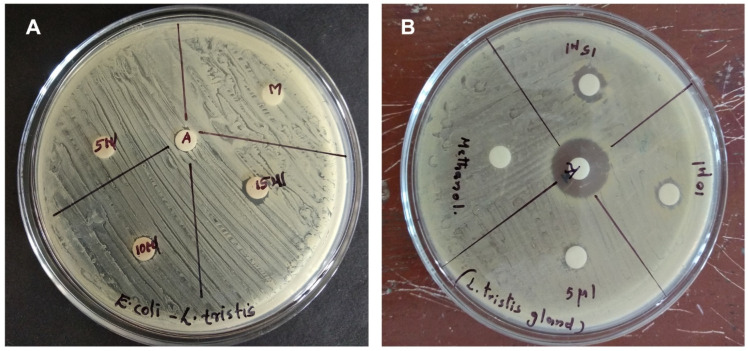
In vitro antibacterial analysis of *L. tristis* defensive secretion on *E. coli* (**A**) and *S. aureus* (**B**).

In vitro antibacterial activity of the defensive gland secretion was carried out following the agar disc diffusion assay method. Two different bacterial strains were used for the analysis. The results of the disc diffusion assay are presented in Figure 5. The data show that the defensive secretion of *L. tristis* is a very effective antimicrobial agent against the bacteria tested. The zone of inhibition against the bacteria *Staphylococcus aureus* and *Escherichia coli* is found to be 11 mm and 9 mm, respectively (Figure 5). 

### 2.5. Cytotoxicity Assay

**Figure 6:**
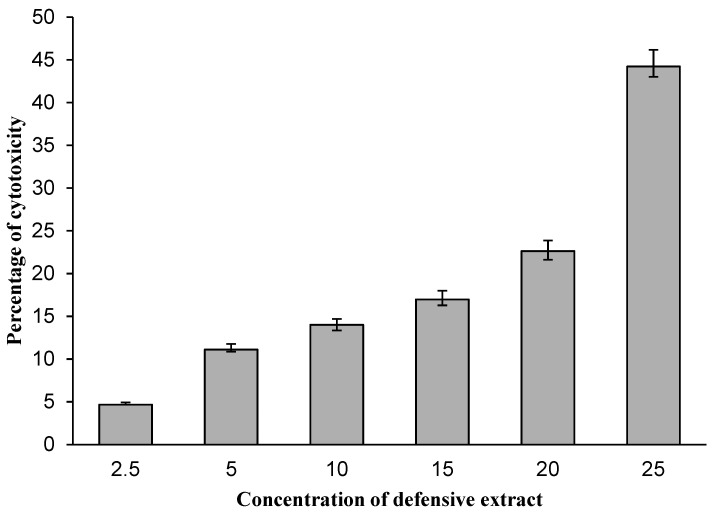
Cytotoxic activity of defensive gland extract of *L. tristis*.

The anticancer property of the defensive gland extract of the *L. tristis* beetle was estimated, and the result of the analysis is presented in Figure 6. A cytotoxicity assay using Dalton’s lymphoma ascites (DLA) cells revealed the anticancer property of the defensive secretion of *L. tristis*. A gradual increase in cytotoxicity was observed with an increase in the concentration (10 µL–200 µL) of the extract. 

## 3. Discussion

The current investigation into the chemical composition of the defensive gland secretion of *L. tristis* showed the presence of 2,3-dimethyl-1,4-benzoquinone, 1,3-dihydroxy-2-methylbenzene, 2,5-dimethylhydroquinone, tetracosane, oleic acid, hexacosane, pentacosane, 7-hexadecenal and tert-hexadecanethiol. Quinone compounds are one of the major biomolecules present in the defensive secretion of beetles [9,10,11]. Earlier studies by Klaus and Thomas (1987) demonstrated two quinone chemicals—methyl-p-benzoquinone and ethyl-p-benzoquinone—which are important components of the defensive secretion of the tenebrionid beetle, *Blaps mucronata* L. They also identified quinone molecules in the defensive gland which induced restlessness in other insects [12]. The significant characteristic of the hydroquinone molecule is its ability to irritate the mucous membranes, skin and eyes of human beings. It is moderately harmful when ingested or absorbed through the skin. Therefore, it is inferred that the quinones and polyphenolic molecules (2,5-dimethyl hydroquinone, 2,3,dimethyl-1,4-benzoquinone and 1,3-dihydroxy-2-methylbenzene) present in the defensive secretion of *L. tristis* are responsible for the skin blistering and irritation in people who are exposed to it. Sonja et al. (2013) analysed the biochemical components in the defensive secretions of three ground beetles—*Abax parallelepipedus* (Piller and Mitter pacher; 1783), *Calosoma sycophanta* L. and *Carabus ullrichii* Gerner—and showed all the three samples contained methacrylic, tiglic and isobutyric acids [13]. Analysis of the chemical components of the defensive gland of both land and water bombardier beetles shows they possess hot quinones, while adephagans contain weak and strong alkaloids, steroids, phenols, carboxylic acids and terpenes [14]. The GC-MS analysis of the defensive secretion of the tenebrionid beetle indicates the presence of toluquinone, ethylquinone and a relatively lower quantity of benzoquinone [4]. In addition to quinones, previously unidentified 4-methyl ketones and unsaturated ketones also are identified in the defensive secretion [5]. The *L. tristis* beetle—a member of the Tenebrionidae family—also contains similar types of quinone chemicals. However, we were unable to identify ketone molecules (4-methyl ketones) in the defensive secretion. Pentacosane and heptacosane are the other chemicals found in the defensive gland extract of *L. tristis*, and these hydrocarbons have also been found in the sting glands of the Braconid wasp, *Bracon hebetor* Say [15]. The hydrocarbon tetracosane, identified in *L. tristis*, was identified in the cuticle of the parasite butterfly *Phengaris nausithous* (Bergstrasser, 1779) [16]. Another significant molecule discovered in *L. tristis* is oleic acid, which is also present in the defensive secretion gland system-1 of the rove beetle, *Deleaster dichrous* (Gravenhorst, 1802) [17]. The sex pheromone 7-hexadecenal, which was isolated in the ovipositor of the female *Heliothis virescens* Fabricius, was discovered in the defensive gland of *L. tristis*. However, it needs to be confirmed experimentally that the chemical acts as a sex pheromone in *L. tristis*. Comparable to the above, the defensive extract of *D. dichrous* has been shown to contain the molecules, sec-butyl decanoate, sec-butyl dodecanoate, sec-butyl (Z)-7-tetradecenoate and isopropyl (Z)-7-tetradecenoate, which are sex pheromone components of the western grape leaf skeletonizer, *Harrisina brillians* (Barnes and McDunnough, 1910). This suggests that the defensive gland secretions act as pheromones or precursors of pheromones in insects [18].

The defensive secretion of the Mupli beetle induced chromosomal aberrations in the dividing cells of *A. cepa*. It is assumed that the defensive secretion enters the cells and stimulates various types of chromosomal damage. The mutagenic research demonstrated that certain heteropteran bug secretions tested on the root tip cells of *Allium* induced significant damage during mitotic division. The scent components of the insect also induced chromosomal abnormalities such as disorientation of chromosomes at metaphase due to non-formation of spindles, unequal separation of chromosomes, formation of anaphase bridges, tripolar grouping and unoriented chromosomes [19]. In the current study, various radical scavenging assays (DPPH, ABTS radical scavenging, peroxide scavenging, and ferric reducing antioxidant power) using the defensive secretion of *L. tristis* revealed promising antioxidant activity. Earlier studies by Liu et al. (2012) using the ethanolic whole-body extract of *Holotrichia parallela* Motschulsky showed that the main component responsible for the antioxidant properties of the defensive gland extracts may be the presence of quinone and phenolic compounds such as methyl-p-benzoquinone and ethyl-p-benzoquinone [20]. We also isolated quinone and phenolic compounds, 2,3-dimethyl-1,4-benzoquinone, 1,3-dihydroxy-2-methylbenzene, 2,5-dimethylhydroquinone from the *L. tristis* defensive gland extract, which may be the reasonable cause of improved antiradical scavenging activity. Earlier research works also studied the antioxidant properties of different extracts of the insects, *Tenebrio molitor* L and *Gryllus bimaculatus* (de Geer) [21,22,23]. 

Compounds with a quinone group are known to induce a variety of physiological actions, including antibacterial, antifungal, antiviral, antimicrobial and anticancer effects [24]. The quinone and polyphenolic molecules present in the defensive extract of *L. tristis* are the probable reason for the antimicrobial activity against the bacteria *E. coli* and *S. aureus*. Similar outcomes were obtained with the millipede *Pachyiulus hungaricus* Karsch (1881) defensive gland extract, which contains high amounts of benzoquinones and hydroquinones and demonstrated substantial antibacterial activity against seven bacterial strains, including *E. coli* and *S. aureus* [25]. Previous studies have revealed that the defensive gland extract from several ground beetle species and the secretions of carrion beetles have antibacterial activities [26]. The pygidial gland secretion of the woodland caterpillar hunter, *Calosoma sycophanta*, was used to identify the antibacterial activities against human bacterial pathogens [10]. Antimicrobial activity of exocrine glandular secretion of *Chrysomela* larvae showed that insect antimicrobial peptide complexes prevent resistance development in bacteria [11]. A cytotoxicity assay using DLA cells revealed the anticancer property of the defensive secretion of *L. tristis*. Similar observations were noticed with the pygidial gland secretions of four ground beetle species (Coleoptera: Carabidae); the results have shown an inhibition of tumour and non-tumour cell proliferation by the antiproliferative effect on the tested cell line [14]. Defensive secretion of *Ulomoides dermestoides* (Fairmaire, 1893) on A549 cells also showed intense cytotoxic activity [27]. Similar to the aforementioned finding, the defensive secretion of *L. tristis* also showed anticancer activity.

## 4. Materials and Methods

### 4.1. Collection of Experimental Organism

The experimental insect *L. tristis* was collected from various localities of Kerala, India, using the hand-picking method from crevices of buildings and rubber plantations. The collected beetles were kept in perforated plastic containers with lids so as to ensure the availability of proper air, temperature and humidity. They were then brought to the laboratory for the extraction of the defensive gland secretion.

### 4.2. Collection of Defensive Secretion from the Gland

Insects of both sexes were used for milking the defensive secretion. The beetles were held between the thumb and index finger after displacing and exposing the elytra and terga and then the defensive gland was located. The terminal part of the abdomen was cleaned with cotton soaked in deionized water. A thin needle was used to irritate the beetle, and the defensive glands were extruded out by gently pressing the abdomen. Much care was taken to extrude the gland out and prevent cross-contamination from other substances such as faecal matter. The tip of the extruded gland was immersed in a solvent contained in an Eppendorf tube (500 µL capacity), and then the gland was broken with a sharp needle. The extract was collected in the Eppendorf tubes containing 300 µL of methanol (HPLC grade) for GC-HRMS analysis. Deionized water was used to collect the extract for the DPPH assay, antimicrobial assay and cytotoxicity experiments. The collected extract was centrifuged at 5000 rpm for 2 min to get rid of any remaining tissue debris. The filtrate was then used for further analysis.

### 4.3. GC-HRMS Analysis

The chemical composition of the defensive gland secretion of the beetle was analysed using a gas chromatograph coupled with mass spectrometer (Jeol, AccuTOF GCV, Agilent) [28]. The methanolic extract of the defensive secretion was used for GC-HRMS analysis. (GC-MS data is available in Supplementary Materials). 

### 4.4. Antimitotic Activity

The antimitotic activity of the defensive extract was examined using healthy, young, uniformly sized *Allium* bulbs. The bulb tissues grown in 300 µL of the defensive secretion were used as experimental groups, and tissues grown in distilled water were used as control groups. The root tips of both the control group and the experimental group were examined using the squash preparation method. The chromosomal aberrations were examined with a microscope (Leica DMi1 inverted microscope) equipped with a camera. The images of the chromosomes were captured at a magnification of 40×. 

### 4.5. In Vitro Antioxidant Activity Analysis

#### 4.5.1. DPPH Radical Scavenging Assay

The antioxidant activity of the extracts was studied by a DPPH assay using ascorbic acid as the standard. Various concentrations of the defensive gland extract were prepared (20 µL, 40 µL, 60 µL, 80 µL and 100 µL) by adding 370 µL DPPH and made up to 2000 µL by adding distilled water. Five different concentrations of ascorbic acid were also prepared. The solutions were kept in the dark for 30 min and analysed using a UV absorption spectrophotometer. 

#### 4.5.2. ABTS Radical Scavenging Assay

The defensive extract of the beetle was mixed with 1 mL of a working solution of the ABTS + radical and incubated for 20 min. The absorbance of each concentration and the control was measured at a wavelength of 734 nm using spectrophotometric analysis [29]. 

#### 4.5.3. Hydrogen Peroxide Scavenging Assay

Different concentrations of the defensive extract of the beetle were mixed with phosphate buffer (0.1 M, pH 7.4) that contained hydrogen peroxide (25 mM). The change in absorbance from the start to the end of 5 min was measured at 230 nm [30].

#### 4.5.4. Ferric Reducing Antioxidant Power

A previous method was used for performing the FRAP experiment. As a FRAP reagent, 300 mM sodium acetate buffer (pH 3.6, 10 mL) was added to 10 mM TPTZ solution in 40 mM hydrochloric acid (1 mL) and 20 mM iron (III) chloride (1 mL). The FRAP reagent was used in a water bath at 37 °C. The sample (20 L) and FRAP reagent (150 L) were mixed, and the absorbance at 593 nm was measured immediately [31].

### 4.6. Antibacterial Activity

In vitro antibacterial activity of the gland extract of the Mupli beetle was studied by the agar disc diffusion assay method. To test the antibacterial activity, *S. aureus* and *E. coli*—two prominent Gram-positive and Gram-negative bacteria—were selected. These pathogens cause various diseases in human beings. The Mueller–Hinton agar (MHA) well diffusion method was employed to investigate the antibacterial activity. Spore suspension of bacteria was added to a sterile Mueller–Hinton medium before solidification. It was then poured into sterile Petri dishes (9 cm in diameter) and spread using a cotton swab. Various concentrations (5 µL, 10 µL and 15 µL) of the defensive gland extract were pipetted into sterile discs of 6 mm and placed at the centre of the Petri dish. The antibiotic kanamycin was used as a positive control in the experiment. The Petri dish was incubated for 16 h at 37 °C. The zone of inhibition was analysed to estimate the antibacterial effect [32].

### 4.7. Anticancer Activity

The gland extract of the beetle was studied for short-term in vitro cytotoxicity using DLA cells. The tumour cells aspirated from the peritoneal cavity of tumour-bearing mice were washed thrice with phosphate-buffered saline (PBS) or normal saline. Cell viability was determined using the trypan blue exclusion method. Viable cell suspension (1 × 10^6^ cells in 0.1 mL) was added to tubes containing various concentrations of the gland extract, and the volume was made up to 1 mL using PBS. The control tube contained only the cell suspension. The assay mixture was incubated for 3 h at 37 °C. After the incubation, the cell suspension was mixed with 0.1 mL of 1% trypan blue, kept for 2–3 min and then loaded on a haemocytometer. Dead cells take up the blue colour of trypan blue, while live cells do not take up the dye. The number of stained and unstained cells was counted individually, and the following formula was used to estimate the percentage of cytotoxicity.

Percentage of cytotoxicity = number of dead cells/number of live cells + number of dead cells × 100 [33].

### 4.8. Statistical Analysis and Data Representation

The results obtained from the cytotoxicity assay were represented as percentage of cytotoxicity, and a Microsoft Excel programme was used to plot the results graphically. The concentration of the sample required to produce 50% scavenging activity (IC_50_) was analysed from the graph through linear regression analysis. The results obtained from the different scavenging assay are represented as percentage ± standard deviation.

## 5. Conclusions

In the current study, the components of the defensive gland extract of the Mupli beetle, *L. tristis*, and its biomedical applications were analysed. The defensive gland extract consists of compounds such as 2,3-dimethyl-1,4-benzoquinone, 1,3-dihydroxy-2-methylbenzene, 2,5-dimethylhydroquinone, tetracosane, oleic acid, hexacosane, pentacosane, 7-hexadecenal and tert-hexadecanethiol. Much effort has been made to find substances from natural sources that can act as effective antimicrobial agents, which has earned great attention as an essential medical need, particularly for pathogenic bacteria. The antibacterial effects of the defensive gland extract of *L. tristis* on the harmful bacteria *S. aureus* and *E. coli* were analysed for the first time in the present study. The defensive gland extract of *L. tristis* also exhibited antioxidant properties. This ability to scavenge free radicals makes this extract advantageous, particularly in illness conditions where there is an abundance of free radical species. The active compounds in the defensive gland extract of *L. tristis* can eliminate these free radicals. The potential anticancer and antimitotic properties of the defensive gland extract imply that the molecules in the beetle are a potentially beneficial source of natural products, and the compounds offer chances for the development of novel chemotherapeutics. In this study, the defensive gland extract of the Mupli beetle, *L. tristis*, which is usually considered a nuisance insect by the human society, was found to have beneficial biochemical properties.

## Figures and Tables

**Table 1:** Compounds in the defensive gland extract of *L. tristis*.

Sl. No	Compound Name	Molecular Formula	Retention Time (min)
1	2,3-dimethyl-1,4-benzoquinone	C_8_H_8_O_2_	5.43
2	1,3-dihydroxy-2-methylbenzene	C_7_H_8_O_2_	8.91
3	2,5-dimethyl hydroquinone	C_8_H_10_O_2_	10.58
4	tetracosane	C_24_H_50_	25.74
5	oleic acid	C_18_H_34_O_2_	26.05
6	hexacosane	C_26_H_54_	29.81
7	pentacosane	C_25_H_52_	31.05
8	7-hexadecenal	C_16_H_30_O	32.31
9	tert-hexadecanethiol	C_16_H_34_S	35.18

**Table 2:** Chromosome aberrations observed in the root meristem cells of *A. cepa* treated with different concentrations of the defensive gland extract of *L. tristis*.

Treatment	Concentration (µL)	Mitotic Index (% ± SD)	Chromosomal Aberration (% ± SD)
Control (distilled water)	100	11.29 ± 0.32	NIL
	200	12.65 ± 0.25	NIL
	300	14.44 ± 0.37	NIL
	400	14.55 ± 0.32	NIL
	500	15.58 ± 0.30	NIL
Experiment	100	9.50 ± 0.50	11.17 ± 0.29
(defensive gland extract)	200	6.57 ± 0.27	13.61 ± 0.27
	300	5.64 ± 0.24	14.46 ± 0.42
	400	3.57 ± 0.36	15.35 ± 0.36
	500	3.33 ± 0.33	16.33 ± 0.33

**Table 3:** The IC_50_ values of antioxidant activities exhibited by the defensive secretion of *L. tristis*.

Sl. No	Assay	IC_50_ Value (µg/mL)
1	DPPH radical scavenging	108.3 ± 2.1
2	ABTS radical scavenging	95.6 ± 1.5
3	H_2_O_2_ scavenging	69.4 ± 3.5
4	Ferric reducing antioxidant power	34.1 ± 1.0

## Data Availability

The data generated and analysed during the current study are available from the corresponding author upon reasonable request.

## Supplementary Materials

The following supporting information can be downloaded at: https://www.mdpi.com/article/10.3390/molecules27217476/s1.
