# Supplementary material for: The Chemical Composition and Antimitotic, Antioxidant, Antibacterial and Cytotoxic Properties of the Defensive Gland Extract of the Beetle, Luprops tristis Fabricius
Source: Molecules. 2022 Nov 2;27(21):7476. doi: 10.3390/molecules27217476 (PMC9657179; doi:10.3390/molecules27217476)
Supplement: Supplementary file 1 [file molecules-27-07476-s001.zip › molecules-1923168-supplementary/GC MS data/GCMS chromatogram.pdf]

Acq. Data Name: E186SANSKRIT_01
Comment: SPLIT1:10-80--8-200-3M-6-275-5M-5-280-EB5...

Ionizing Volt: 70[V]
External Sample Id: 01
Instrument Configuration: JMS-T100GCV

Experiment Date/Time: 2/20/2020 12:54:48 PM
Ionization Mode: EI+
JEOL The AccuTOF GCv: 35.00..650.00

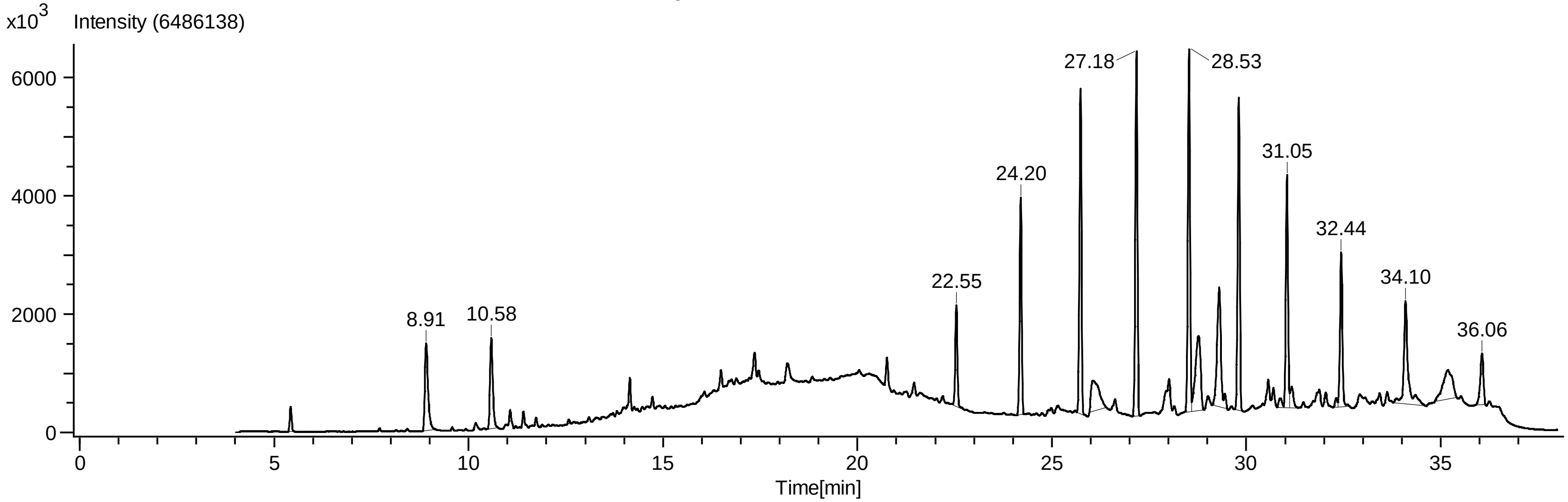

Peak Number	Time [min]	Type	Peak Width(FWH [min]	Area [Intens. * sec]	Height	Description	Start Point		End Point	
							Time[min]	Height	Time[min]	Height
1	5.43	BB	0.0520	1372221.18	417753.59		5.33	13957	5.50	19105
2	8.91	BB	0.0831	8363502.30	1475792.19		8.78	20450	9.14	53116
3	10.58	BB	0.0749	7591079.93	1532743.98		10.44	53650	10.76	79924
4	22.55	BB	0.0593	6665423.53	1710943.08		22.39	480772	22.75	380285
5	24.20	BB	0.0585	13608522.51	3674449.07		24.10	292917	24.28	307242
6	25.74	BB	0.0593	20750685.18	5489156.04		25.64	346657	25.91	269666
7	26.05	BB	0.2511	7539310.21	516188.78		25.96	346408	26.40	417755
8	27.18	BB	0.0607	23938840.52	6171654.31		27.07	267047	27.26	279729
9	28.53	BV	0.0603	23815846.49	6139037.94		28.35	332579	28.60	353240
10	28.77	VB	0.1479	11953684.37	1266707.86		28.60	353240	28.91	379487
11	29.30	BB	0.1007	14209764.84	2013546.36		29.13	462879	29.51	402981
12	29.81	BB	0.0598	20197957.21	5294588.41		29.69	387686	29.94	352370
13	30.88	BV	0.0998	886844.44	147928.16		30.79	427071	30.95	423856
14	31.05	VV	0.0608	15341476.25	3937103.44		30.95	423856	31.11	420769
15	31.17	VB	0.1014	2073482.52	354184.91		31.11	420769	31.33	416525
16	32.31	BV	0.0811	647679.12	154677.15		32.22	423773	32.36	428577
17	32.44	VB	0.0661	11395221.72	2615177.91		32.36	428577	32.66	439383
18	34.10	BB	0.0846	13373258.55	1740620.72		33.77	504172	34.60	453333
19	35.18	BB	0.2789	8312741.74	486847.71		34.84	524367	35.41	594495
20	36.06	BB	0.0855	4884788.37	865047.37		35.88	459278	36.15	476020
