# Supplementary figures and images for: The Chemical Composition and Antimitotic, Antioxidant, Antibacterial and Cytotoxic Properties of the Defensive Gland Extract of the Beetle, Luprops tristis Fabricius
Source: Molecules. 2022 Nov 2;27(21):7476. doi: 10.3390/molecules27217476 (PMC9657179; doi:10.3390/molecules27217476)

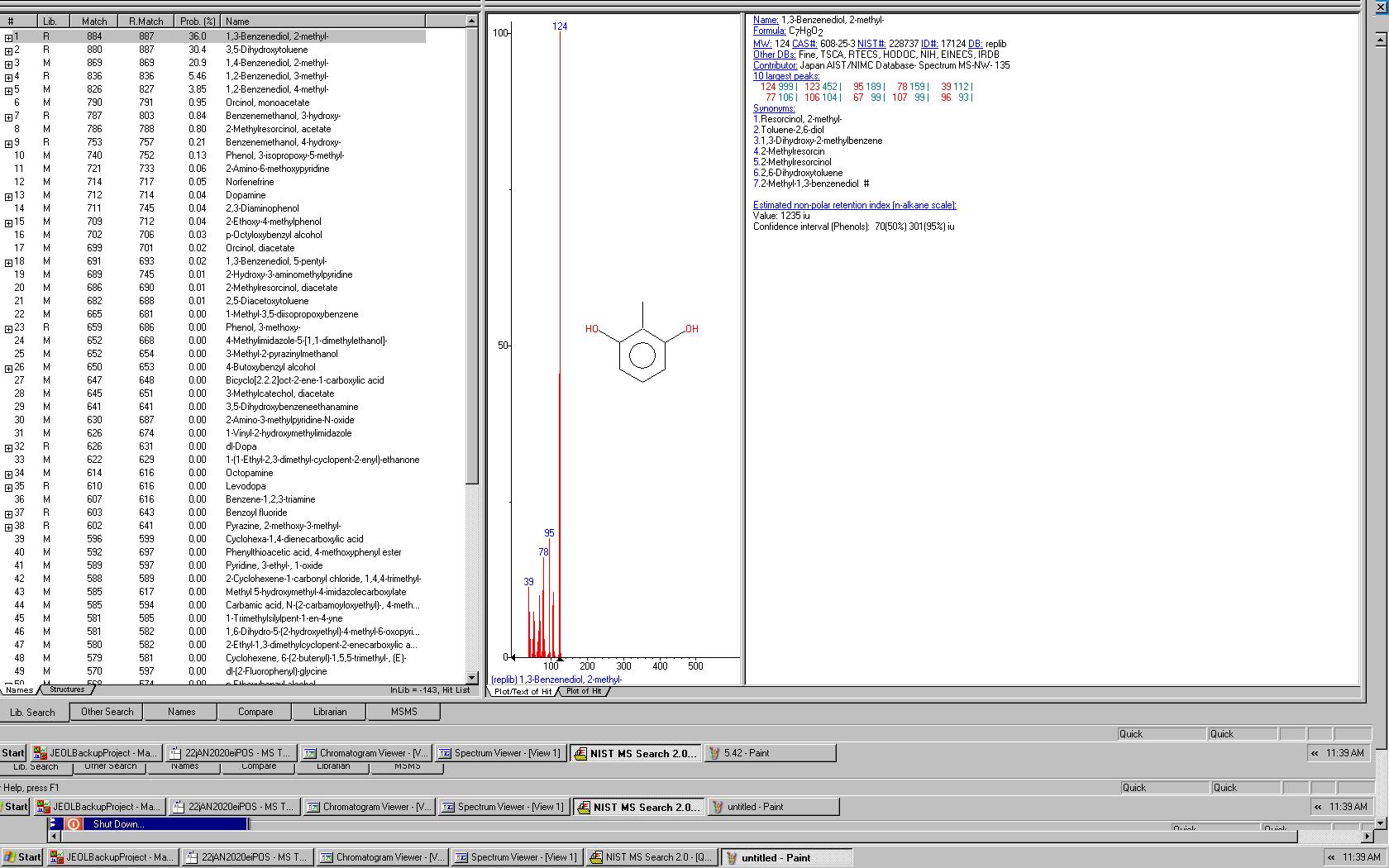

Supplement: Supplementary file 1 [file molecules-27-07476-s001.zip › molecules-1923168-supplementary/GC MS data/GC MS/1,3 dihydroxy 2, methyl benzene.JPG]

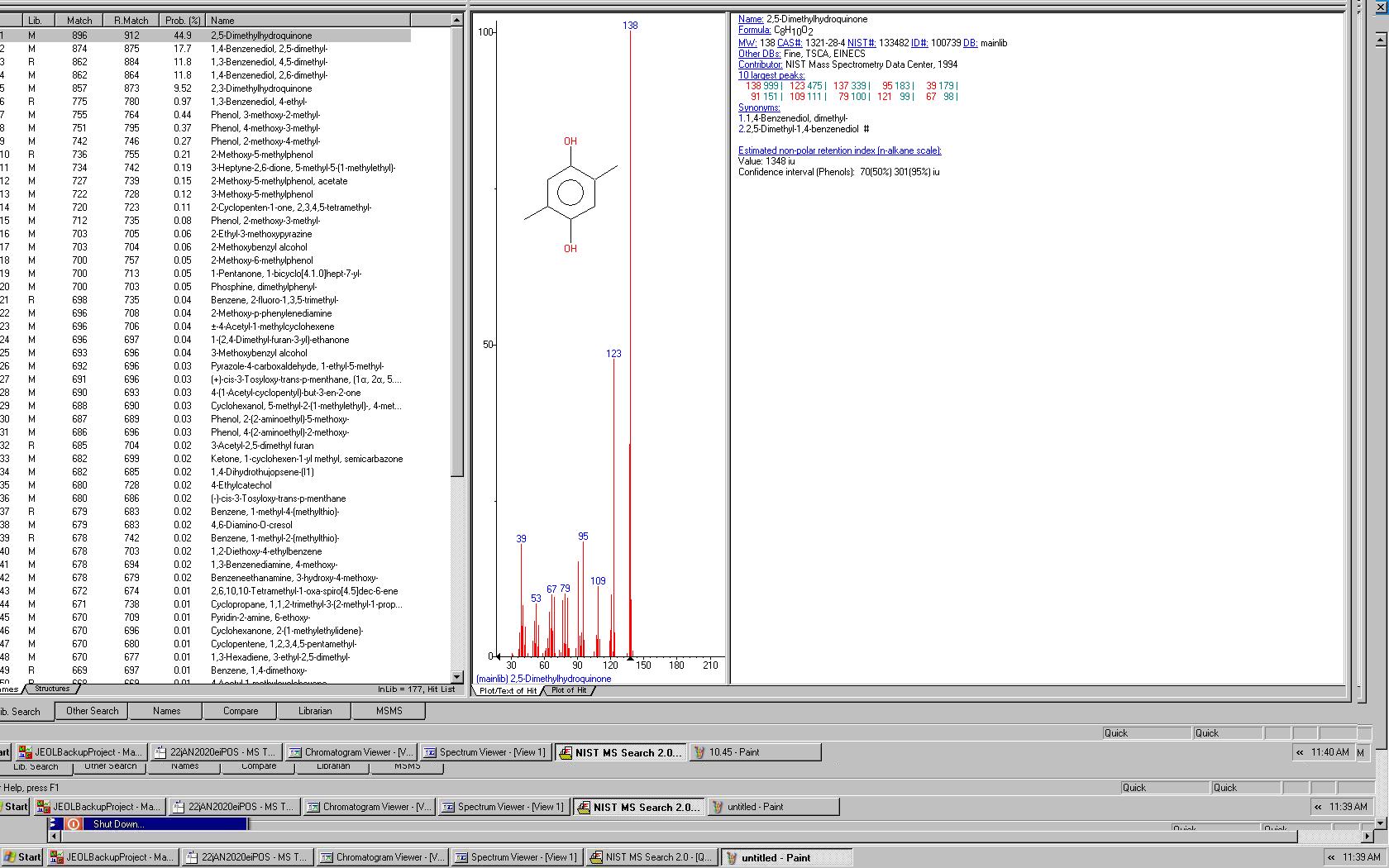

Supplement: Supplementary file 1 [file molecules-27-07476-s001.zip › molecules-1923168-supplementary/GC MS data/GC MS/2,5 dimethyl hydroquinone.JPG]

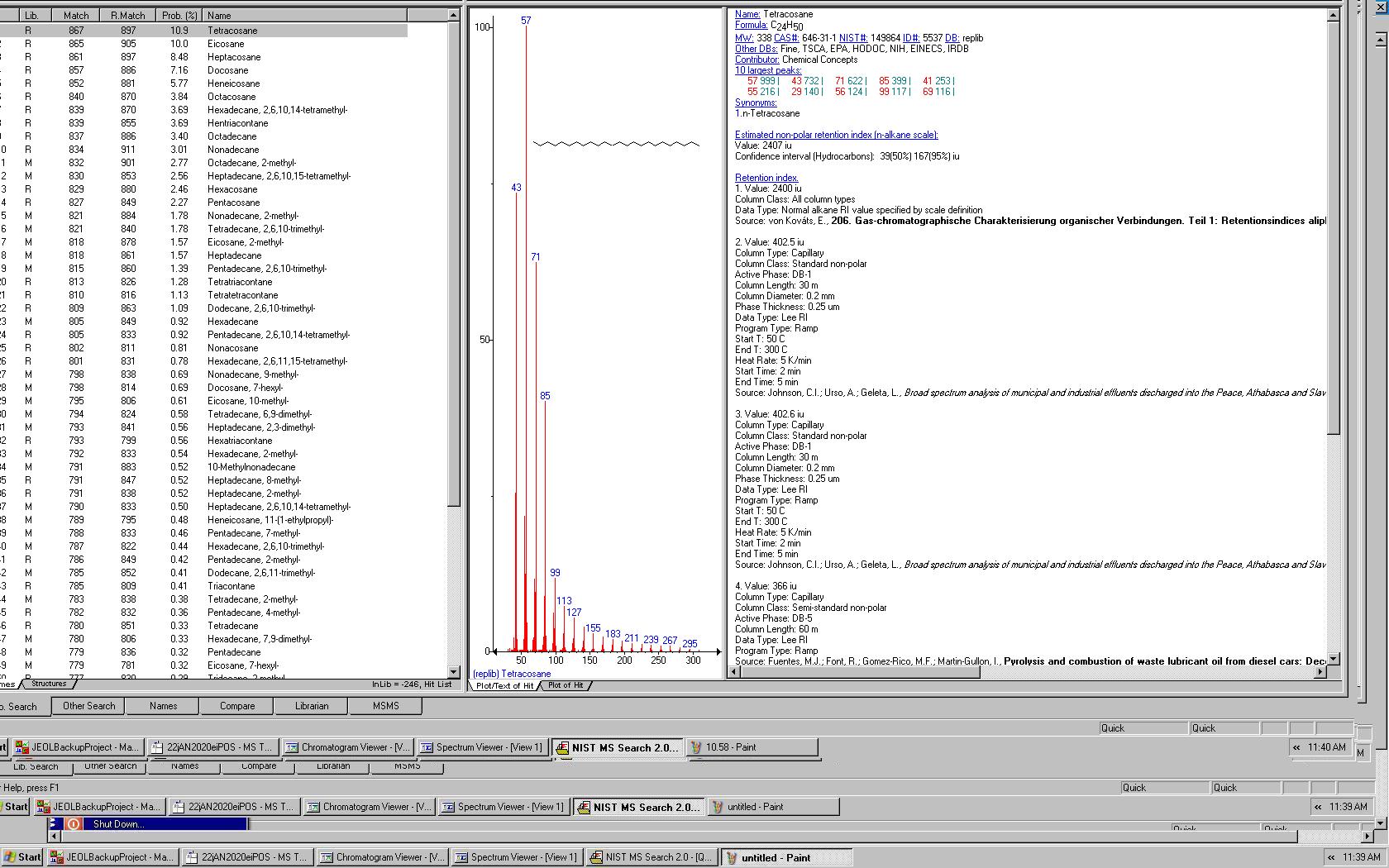

Supplement: Supplementary file 1 [file molecules-27-07476-s001.zip › molecules-1923168-supplementary/GC MS data/GC MS/22.54.JPG]

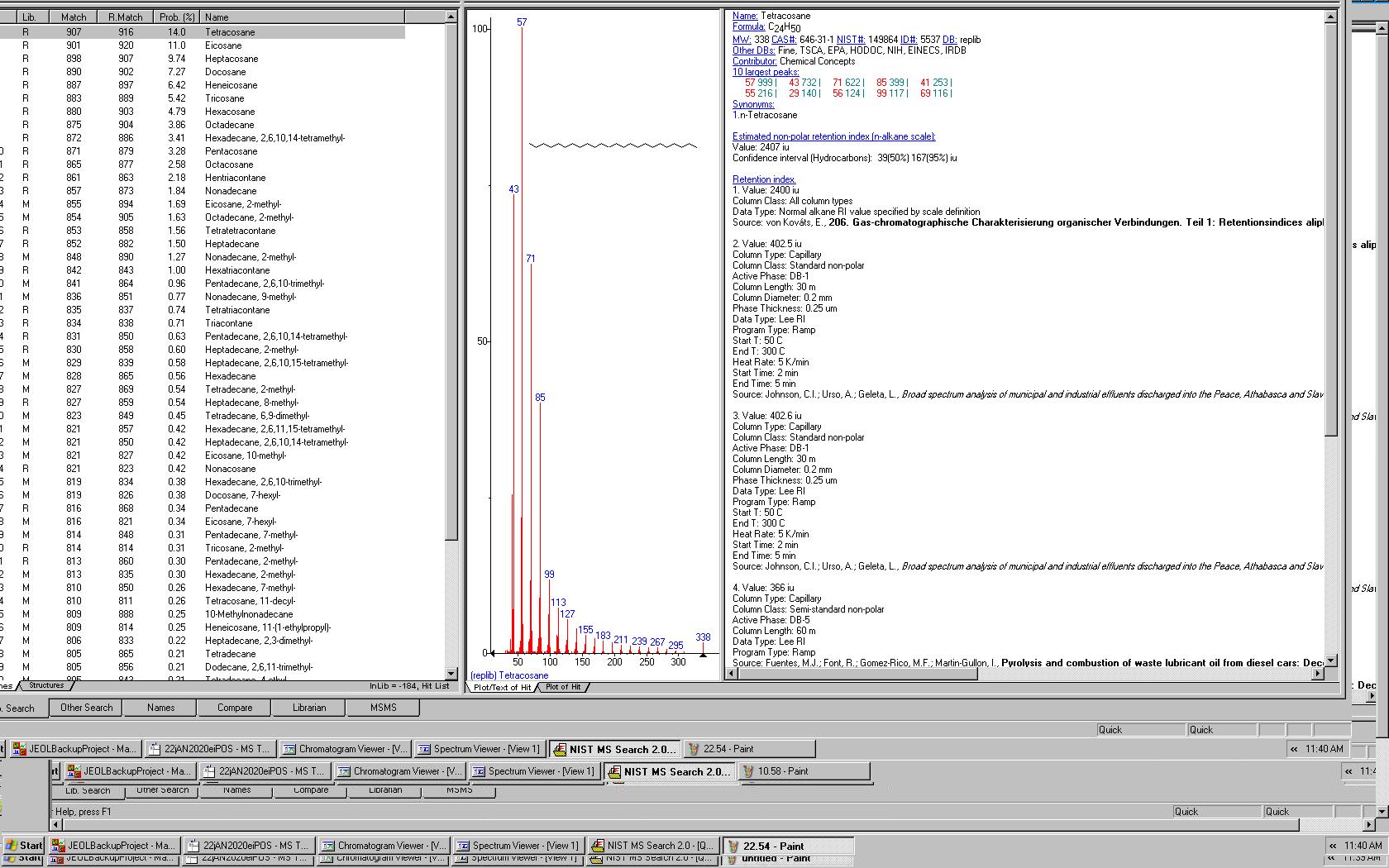

Supplement: Supplementary file 1 [file molecules-27-07476-s001.zip › molecules-1923168-supplementary/GC MS data/GC MS/24.19.JPG]

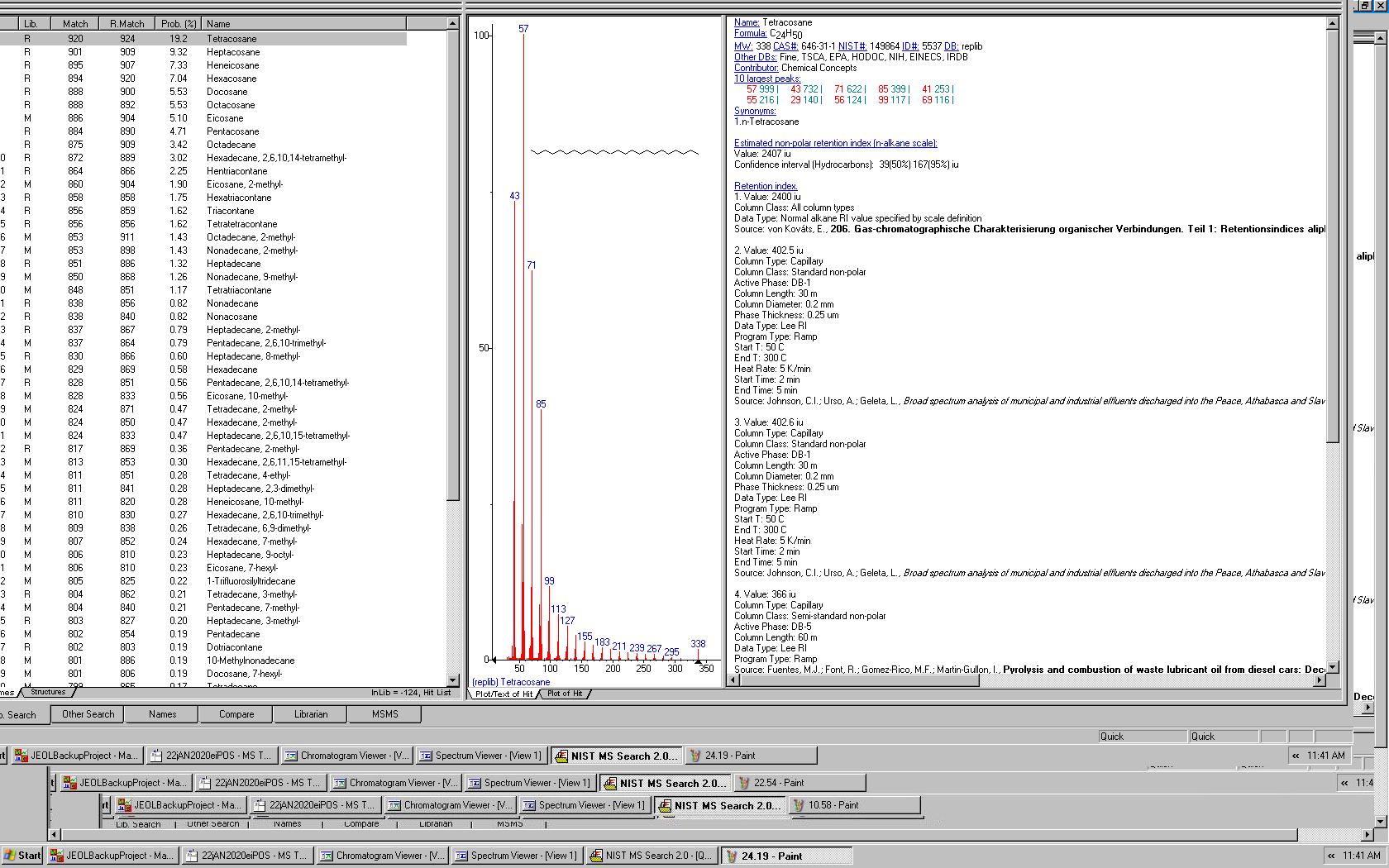

Supplement: Supplementary file 1 [file molecules-27-07476-s001.zip › molecules-1923168-supplementary/GC MS data/GC MS/25.72.JPG]

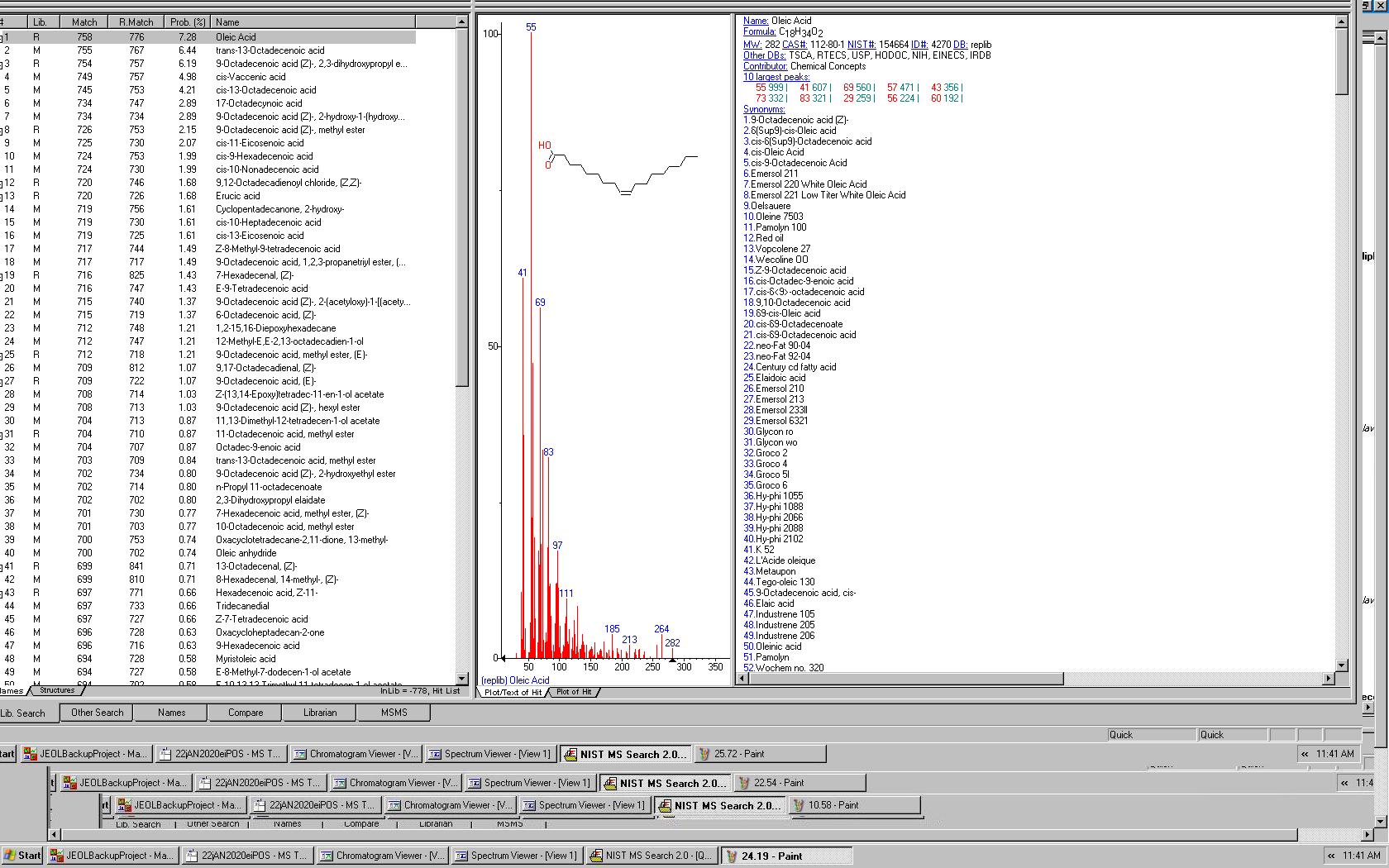

Supplement: Supplementary file 1 [file molecules-27-07476-s001.zip › molecules-1923168-supplementary/GC MS data/GC MS/26.06.JPG]

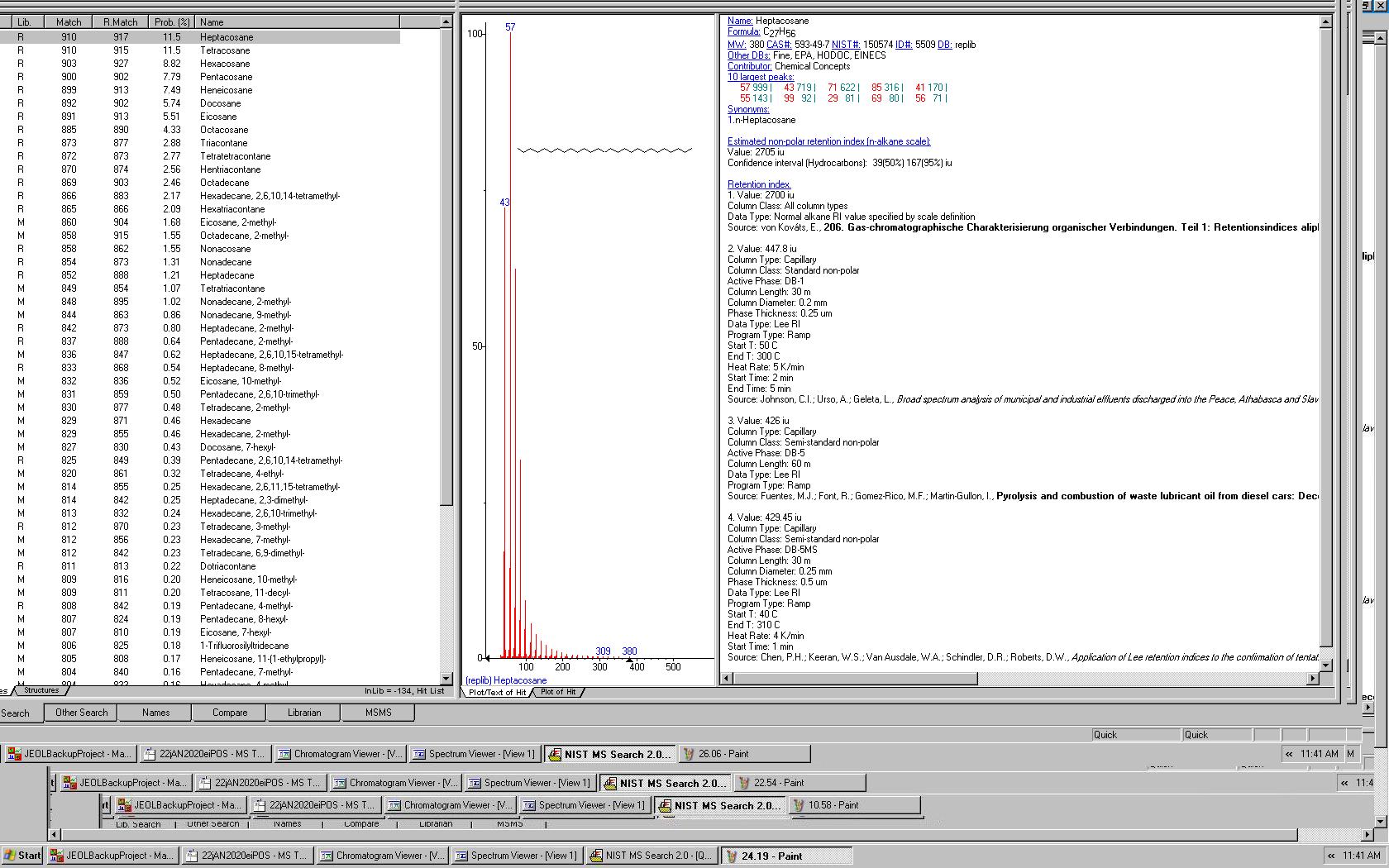

Supplement: Supplementary file 1 [file molecules-27-07476-s001.zip › molecules-1923168-supplementary/GC MS data/GC MS/27.18.JPG]

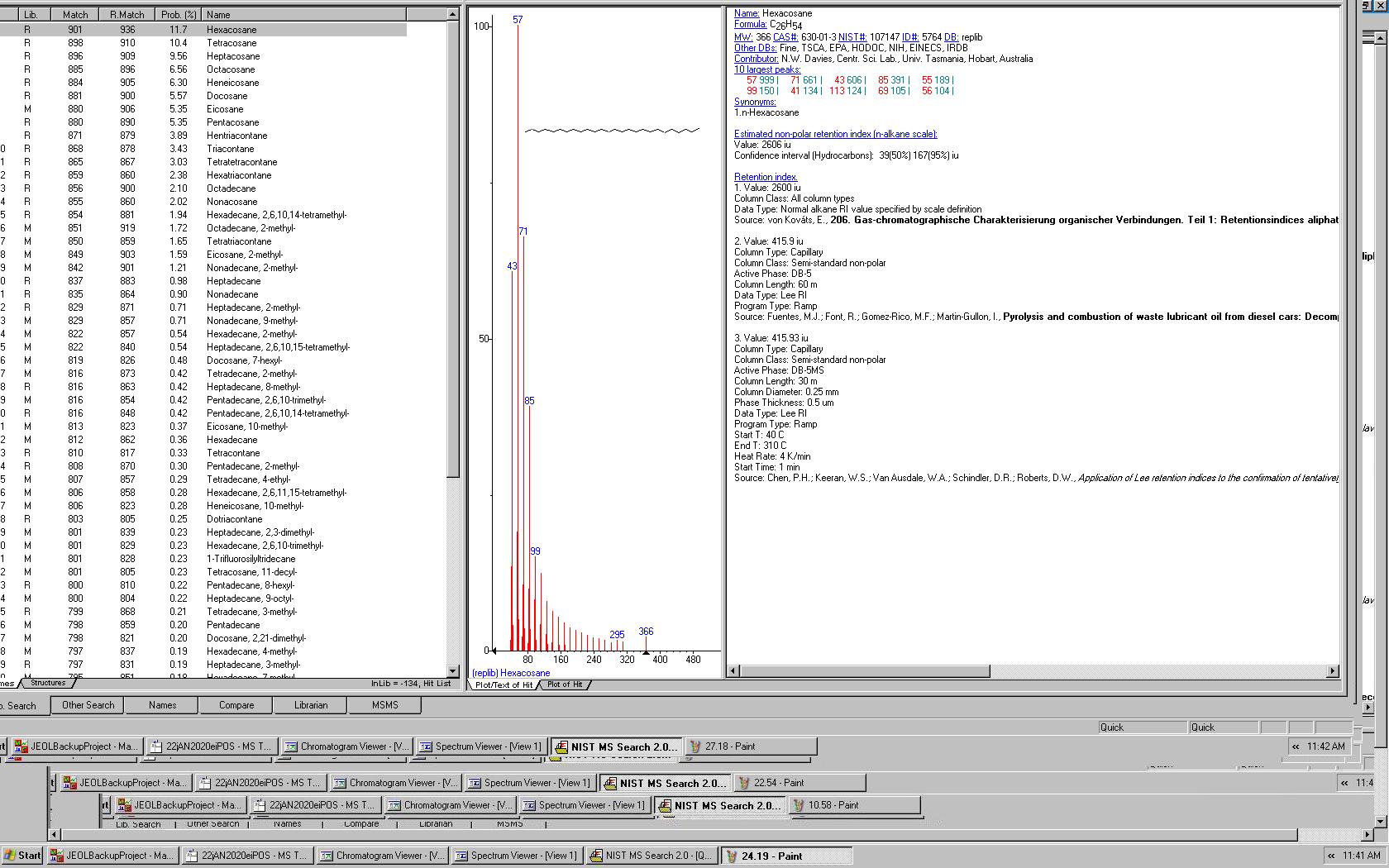

Supplement: Supplementary file 1 [file molecules-27-07476-s001.zip › molecules-1923168-supplementary/GC MS data/GC MS/28.53.JPG]

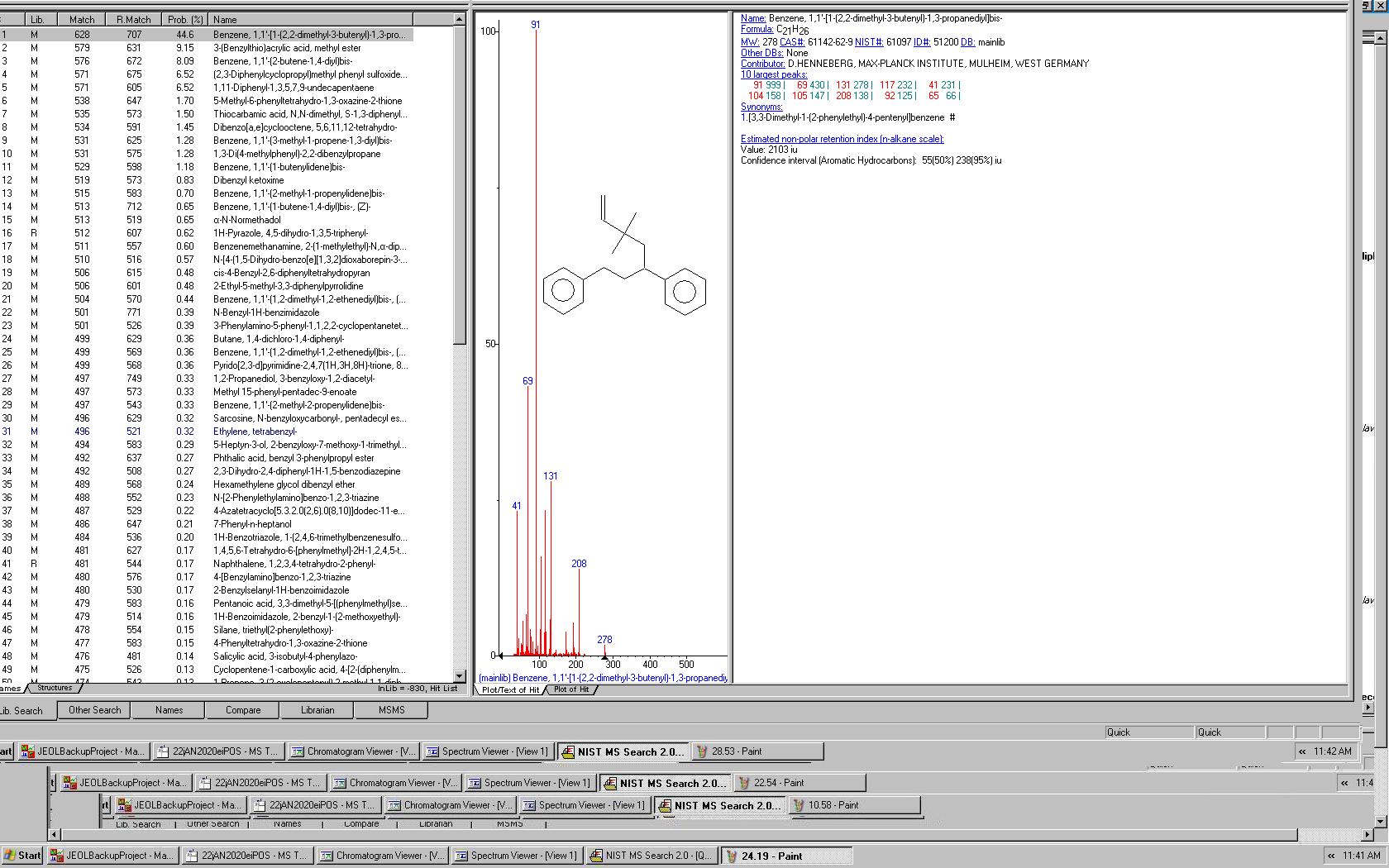

Supplement: Supplementary file 1 [file molecules-27-07476-s001.zip › molecules-1923168-supplementary/GC MS data/GC MS/28.77.JPG]

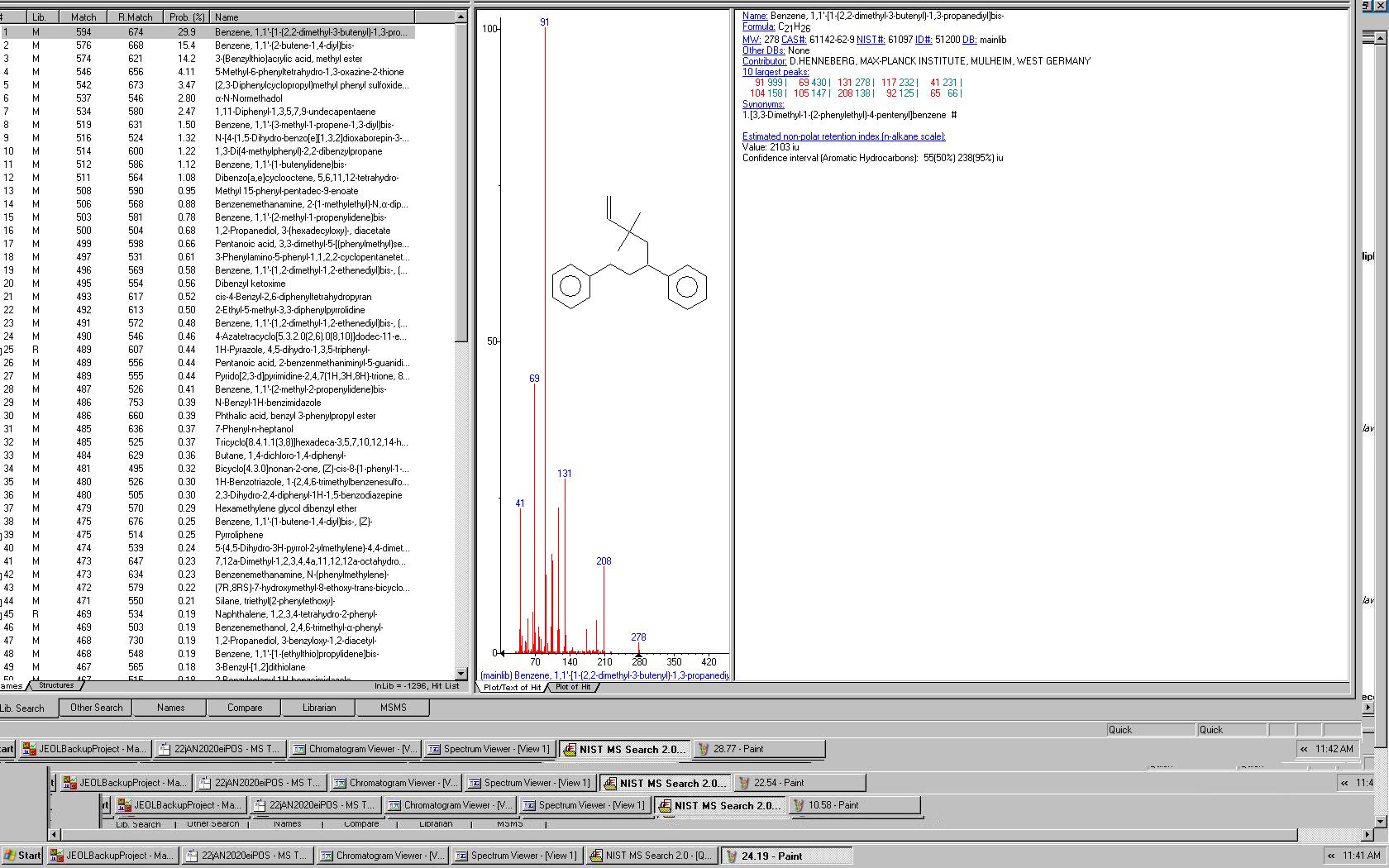

Supplement: Supplementary file 1 [file molecules-27-07476-s001.zip › molecules-1923168-supplementary/GC MS data/GC MS/29.30.JPG]

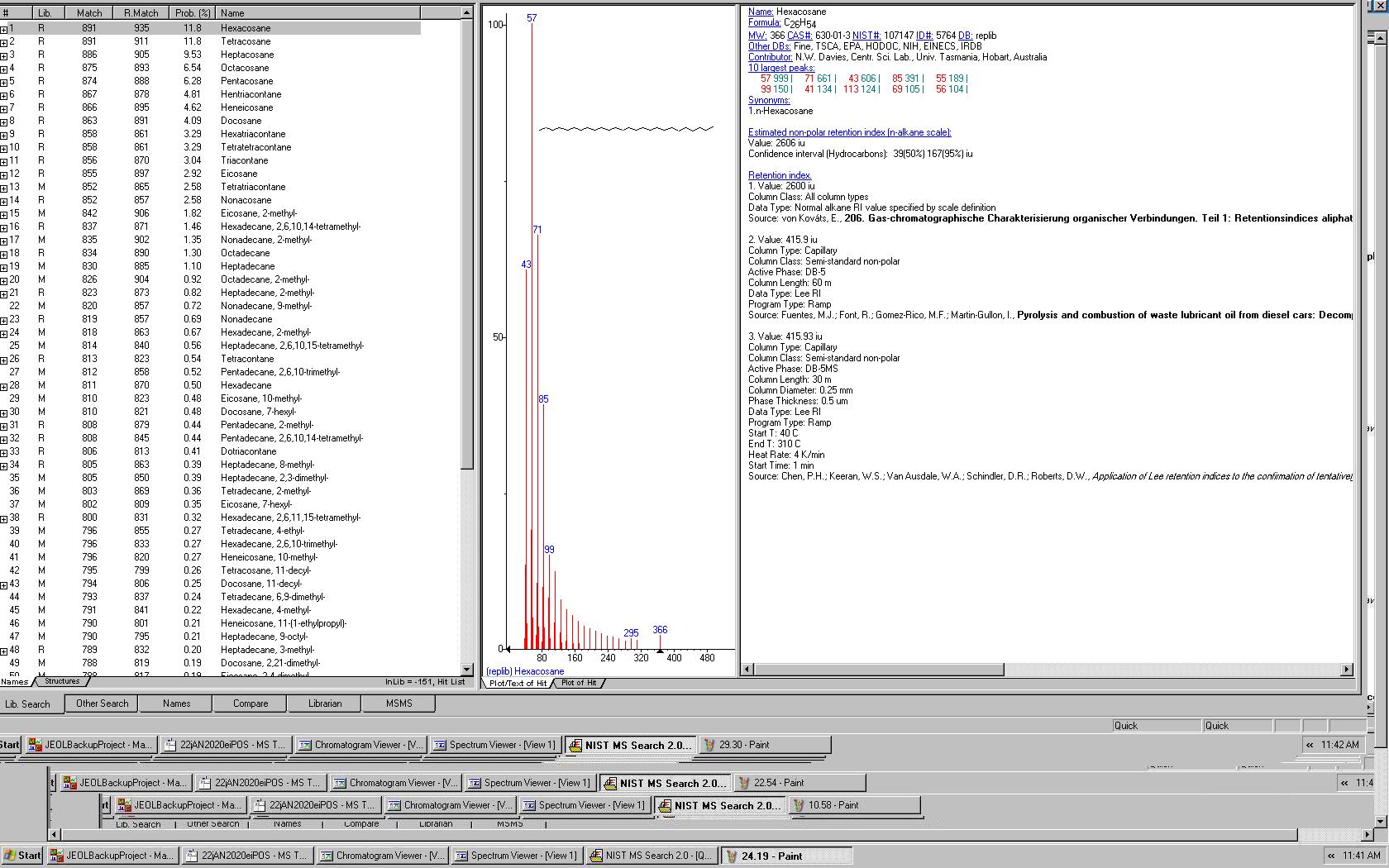

Supplement: Supplementary file 1 [file molecules-27-07476-s001.zip › molecules-1923168-supplementary/GC MS data/GC MS/29.81.JPG]

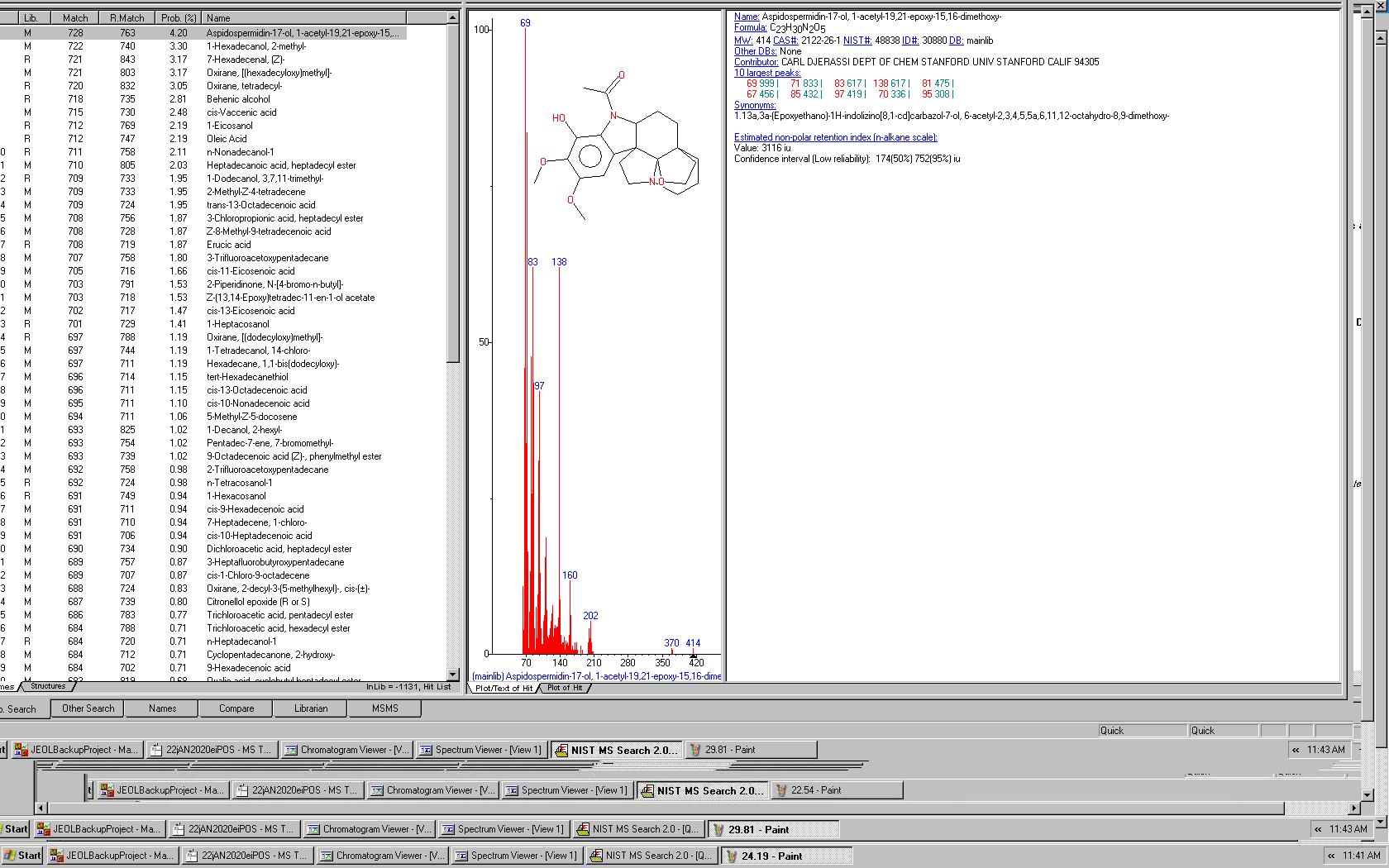

Supplement: Supplementary file 1 [file molecules-27-07476-s001.zip › molecules-1923168-supplementary/GC MS data/GC MS/30.87.JPG]

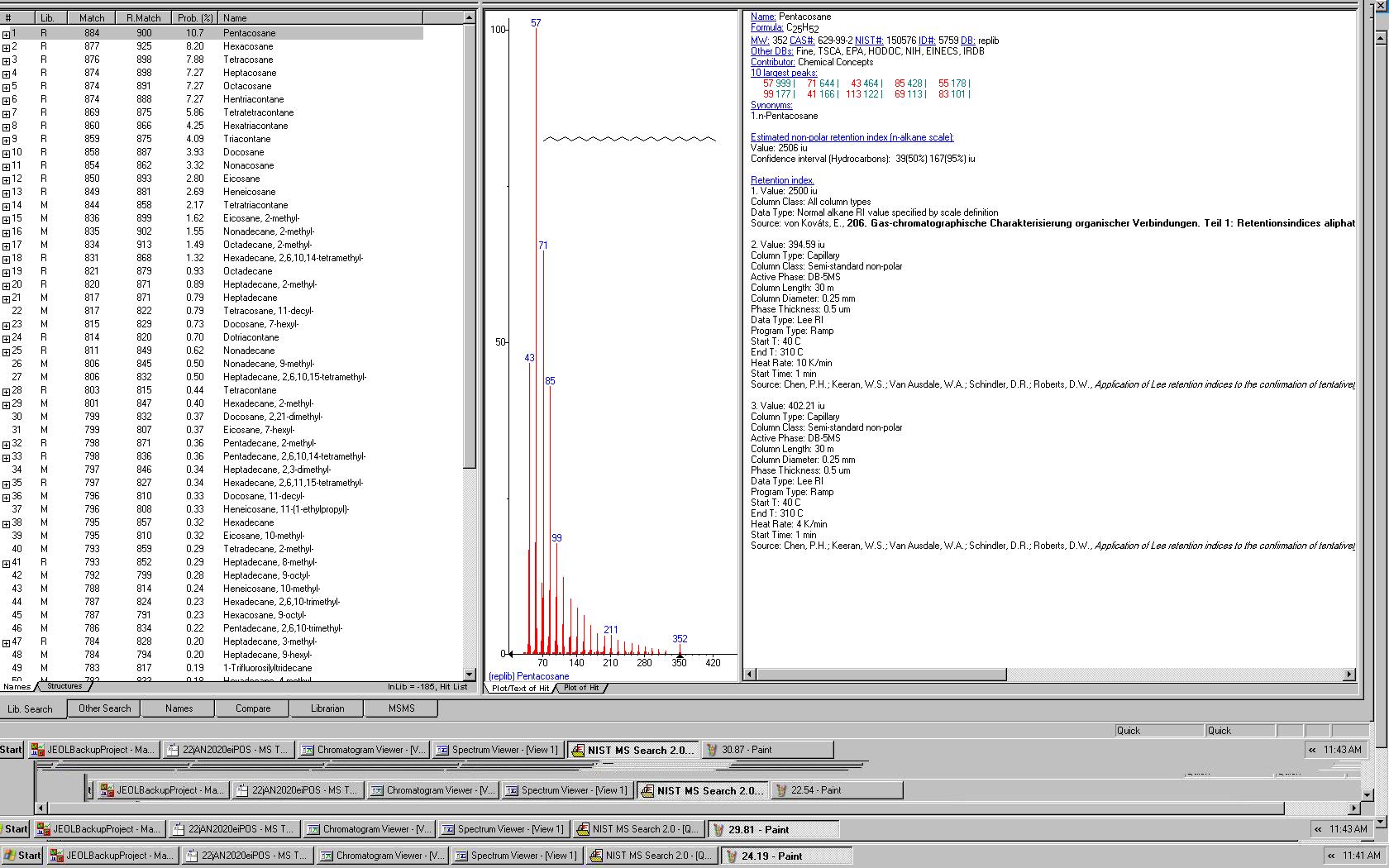

Supplement: Supplementary file 1 [file molecules-27-07476-s001.zip › molecules-1923168-supplementary/GC MS data/GC MS/31.04.JPG]

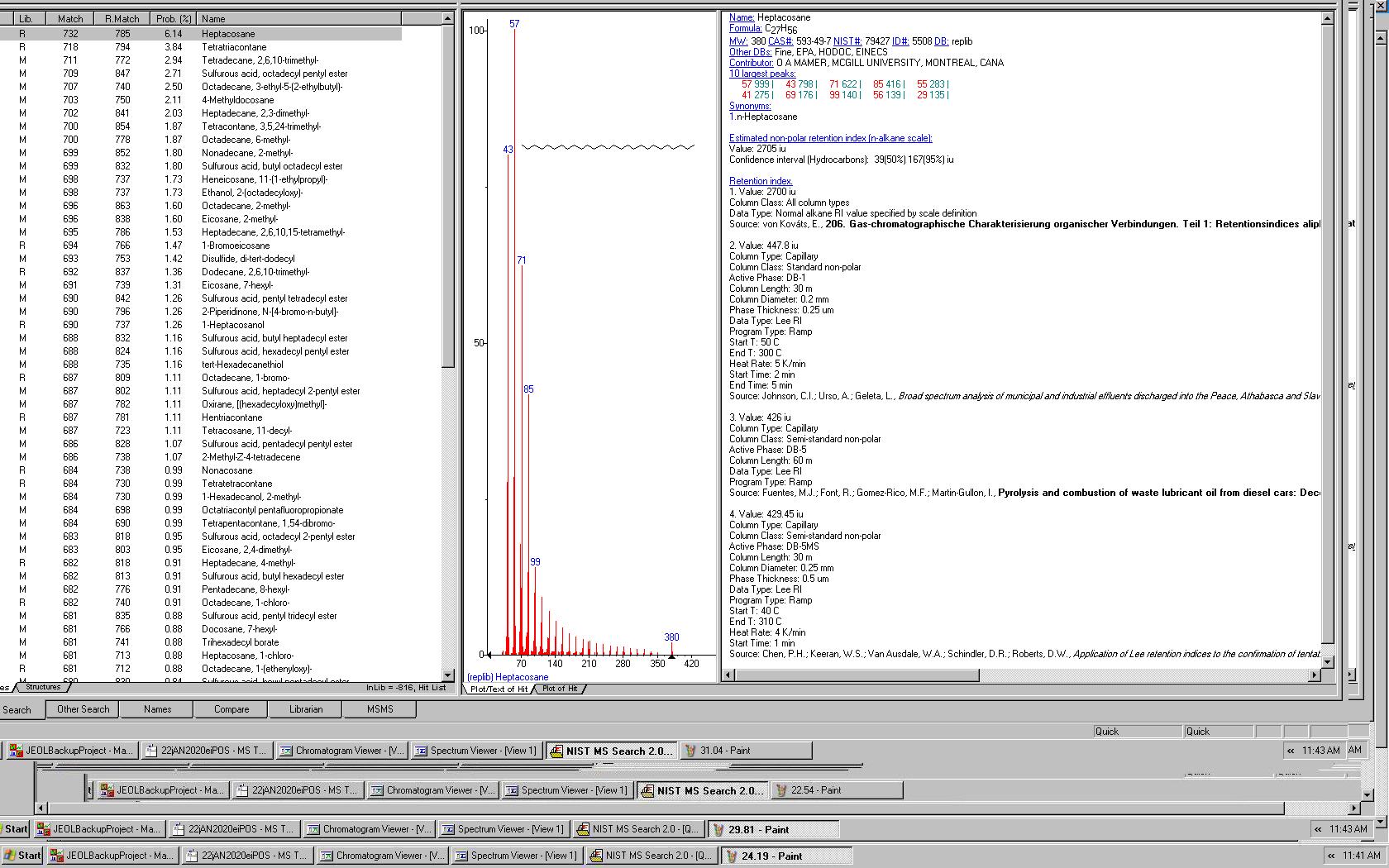

Supplement: Supplementary file 1 [file molecules-27-07476-s001.zip › molecules-1923168-supplementary/GC MS data/GC MS/31.16.JPG]

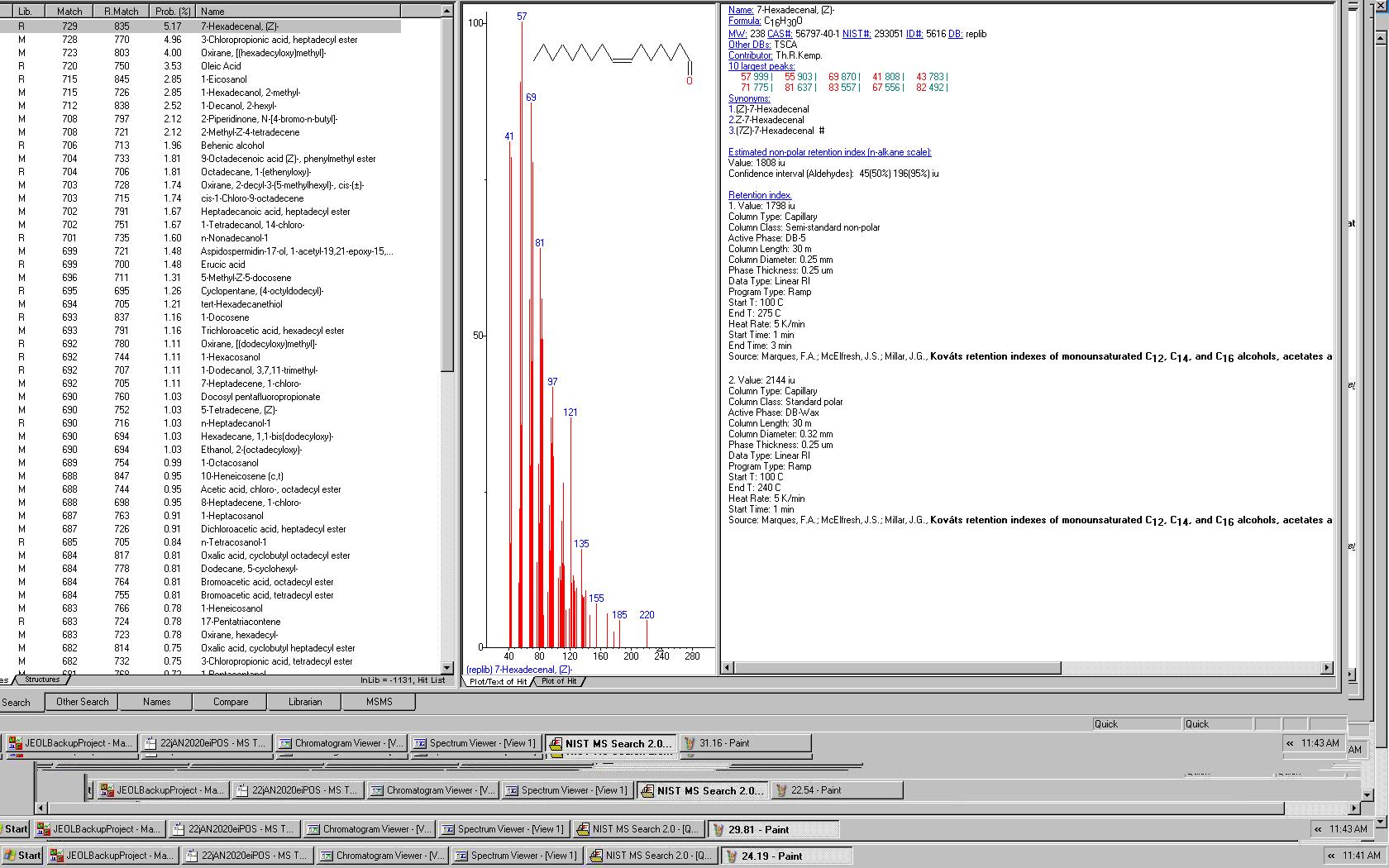

Supplement: Supplementary file 1 [file molecules-27-07476-s001.zip › molecules-1923168-supplementary/GC MS data/GC MS/32.31.JPG]

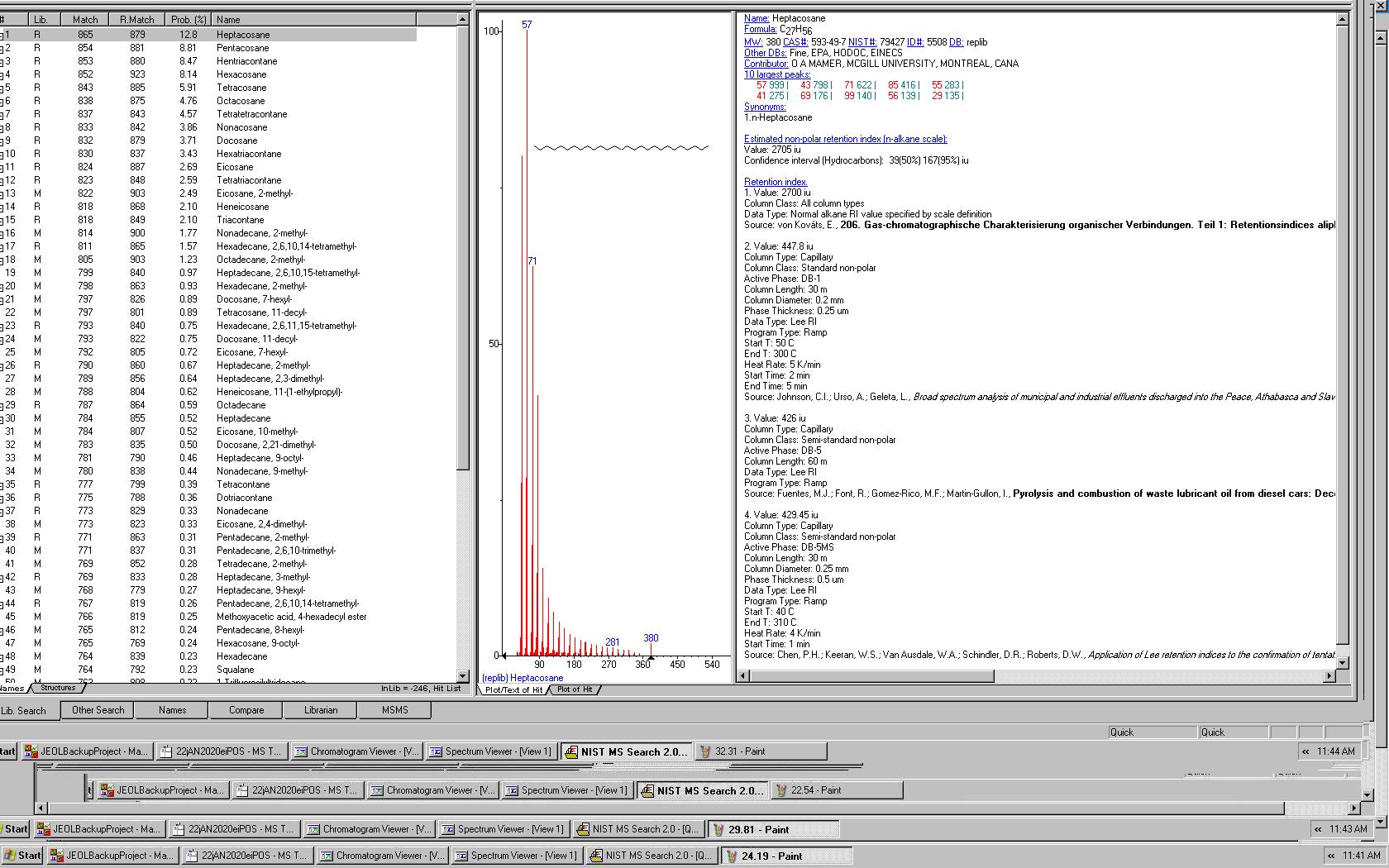

Supplement: Supplementary file 1 [file molecules-27-07476-s001.zip › molecules-1923168-supplementary/GC MS data/GC MS/32.44.JPG]

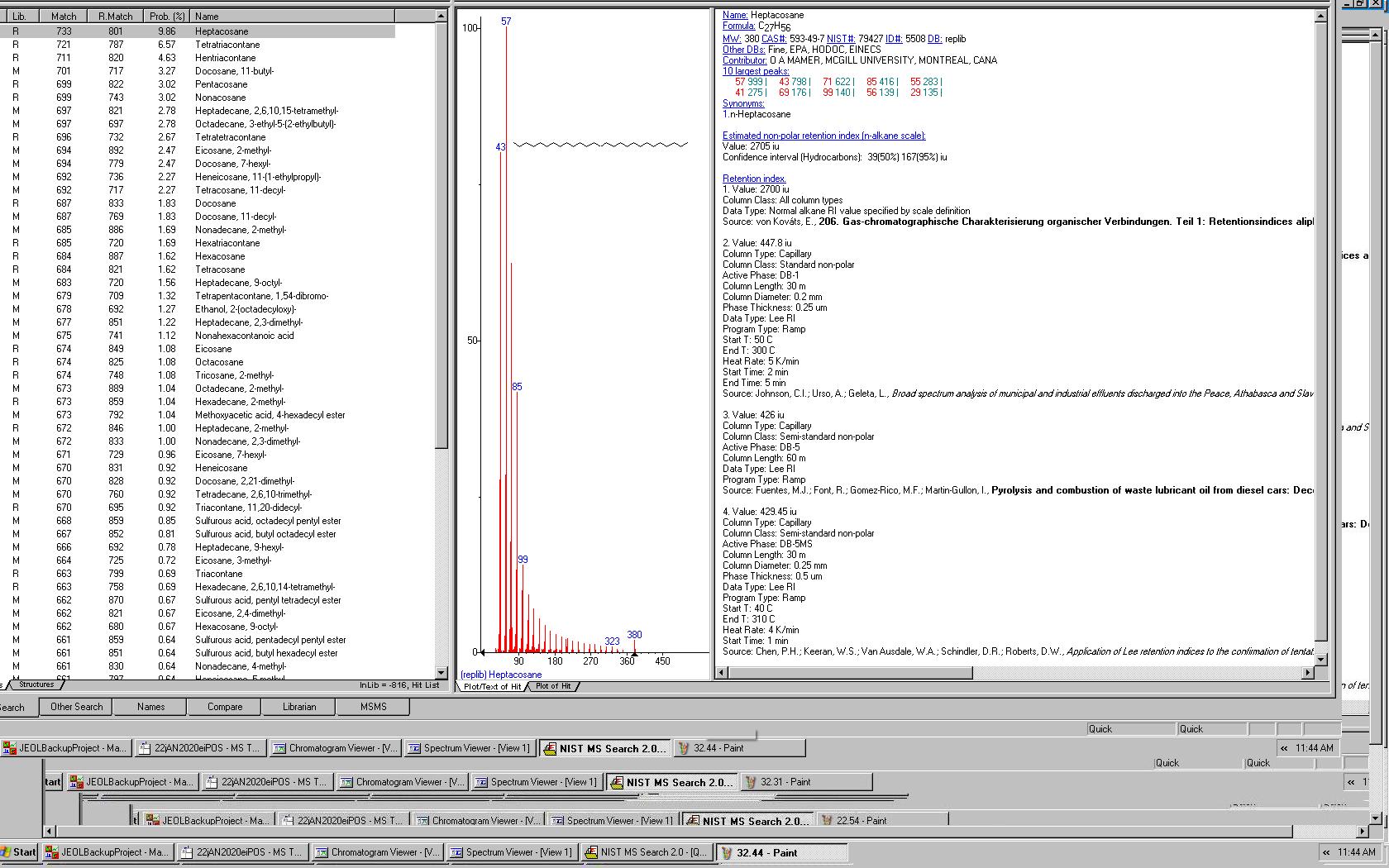

Supplement: Supplementary file 1 [file molecules-27-07476-s001.zip › molecules-1923168-supplementary/GC MS data/GC MS/34.08.JPG]

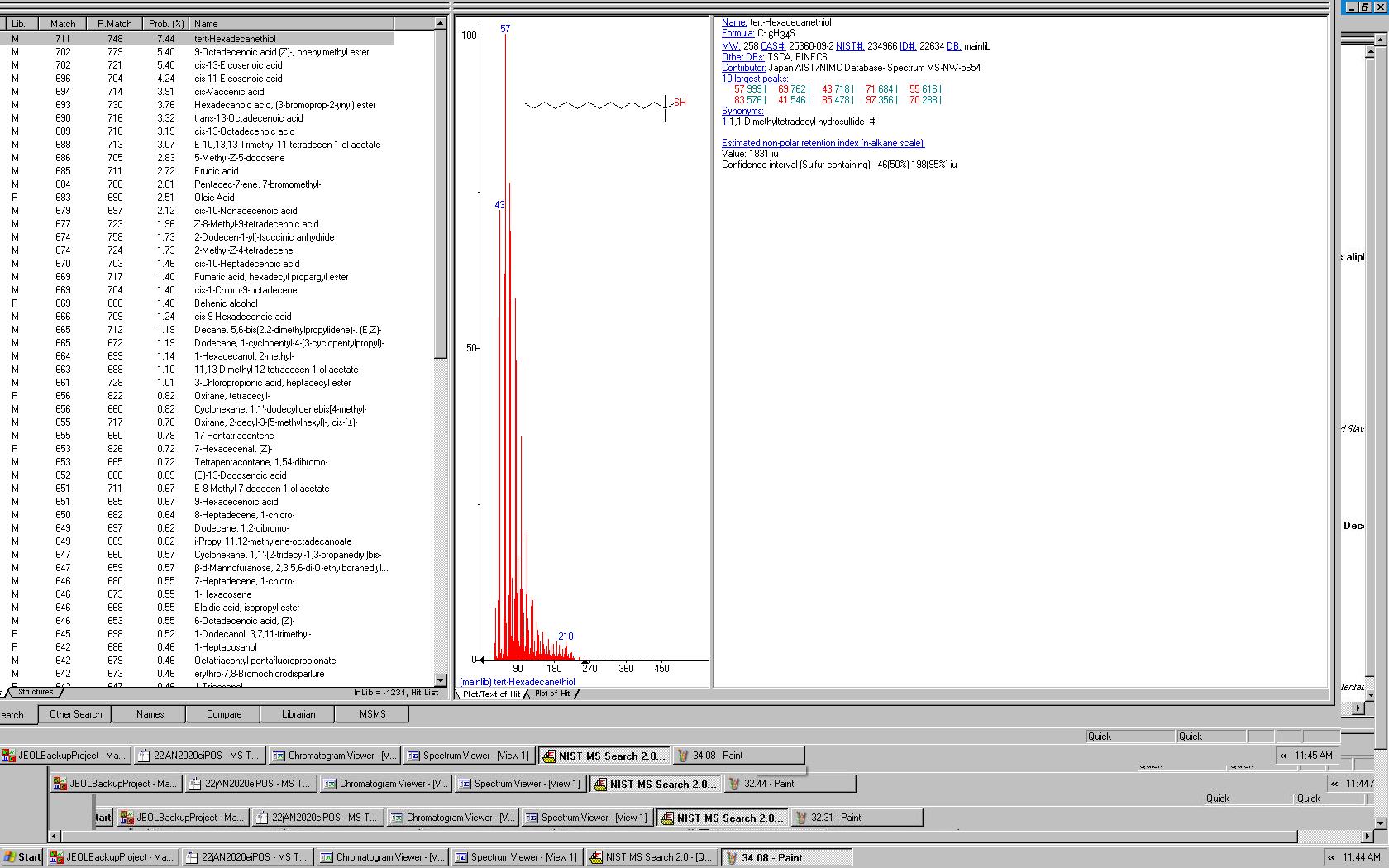

Supplement: Supplementary file 1 [file molecules-27-07476-s001.zip › molecules-1923168-supplementary/GC MS data/GC MS/35.19.JPG]

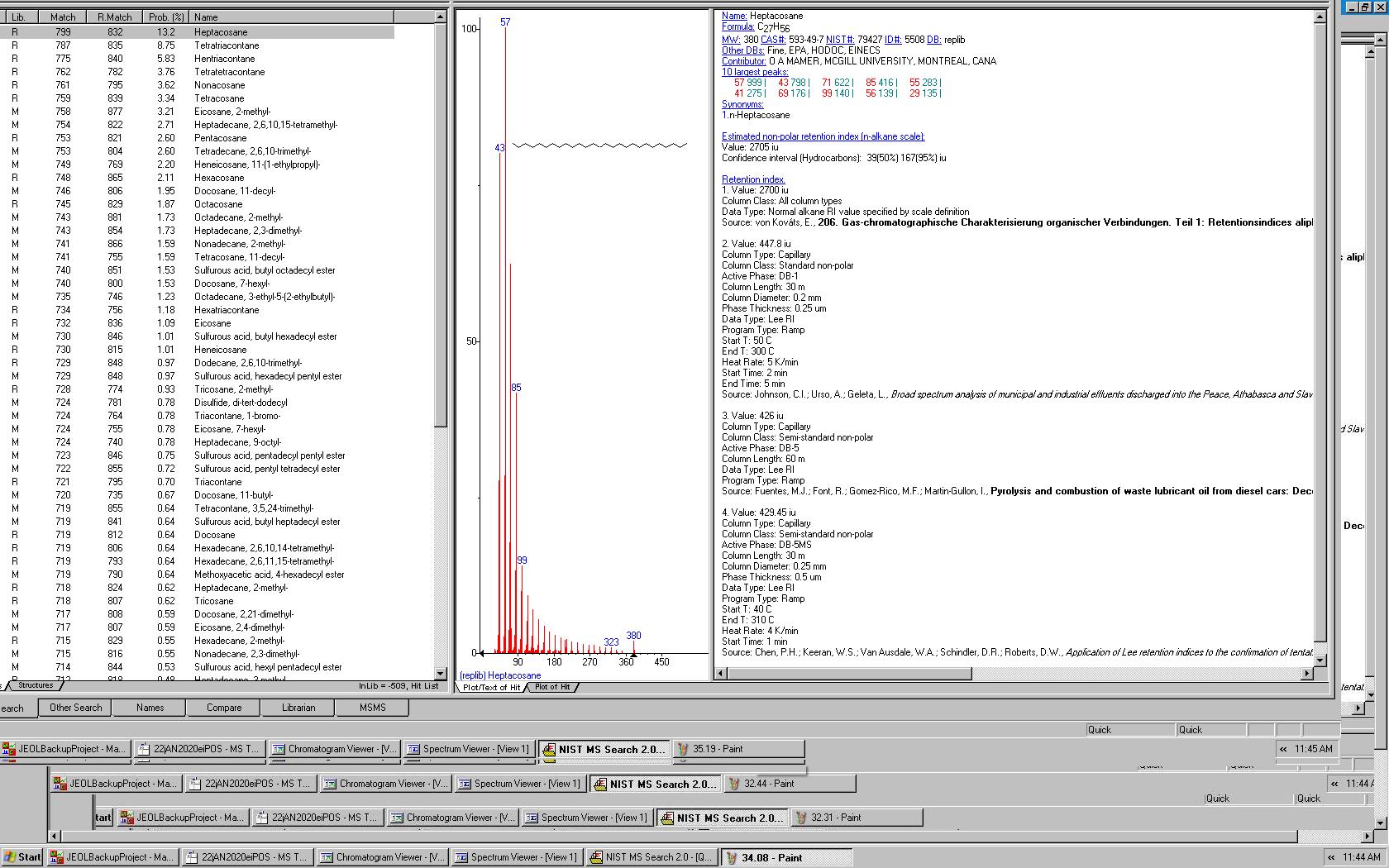

Supplement: Supplementary file 1 [file molecules-27-07476-s001.zip › molecules-1923168-supplementary/GC MS data/GC MS/36.05.JPG]

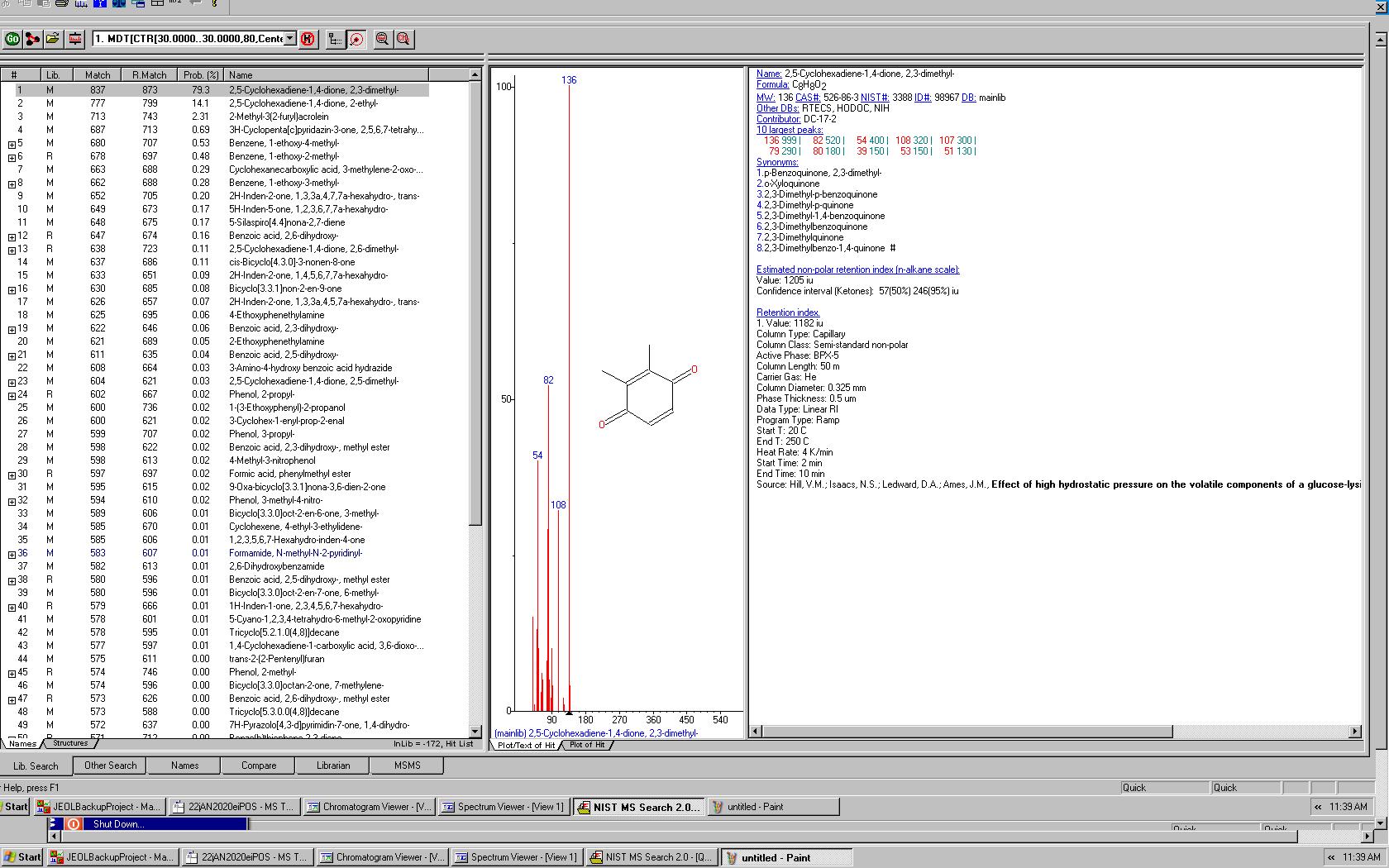

Supplement: Supplementary file 1 [file molecules-27-07476-s001.zip › molecules-1923168-supplementary/GC MS data/GC MS/5.42.JPG]

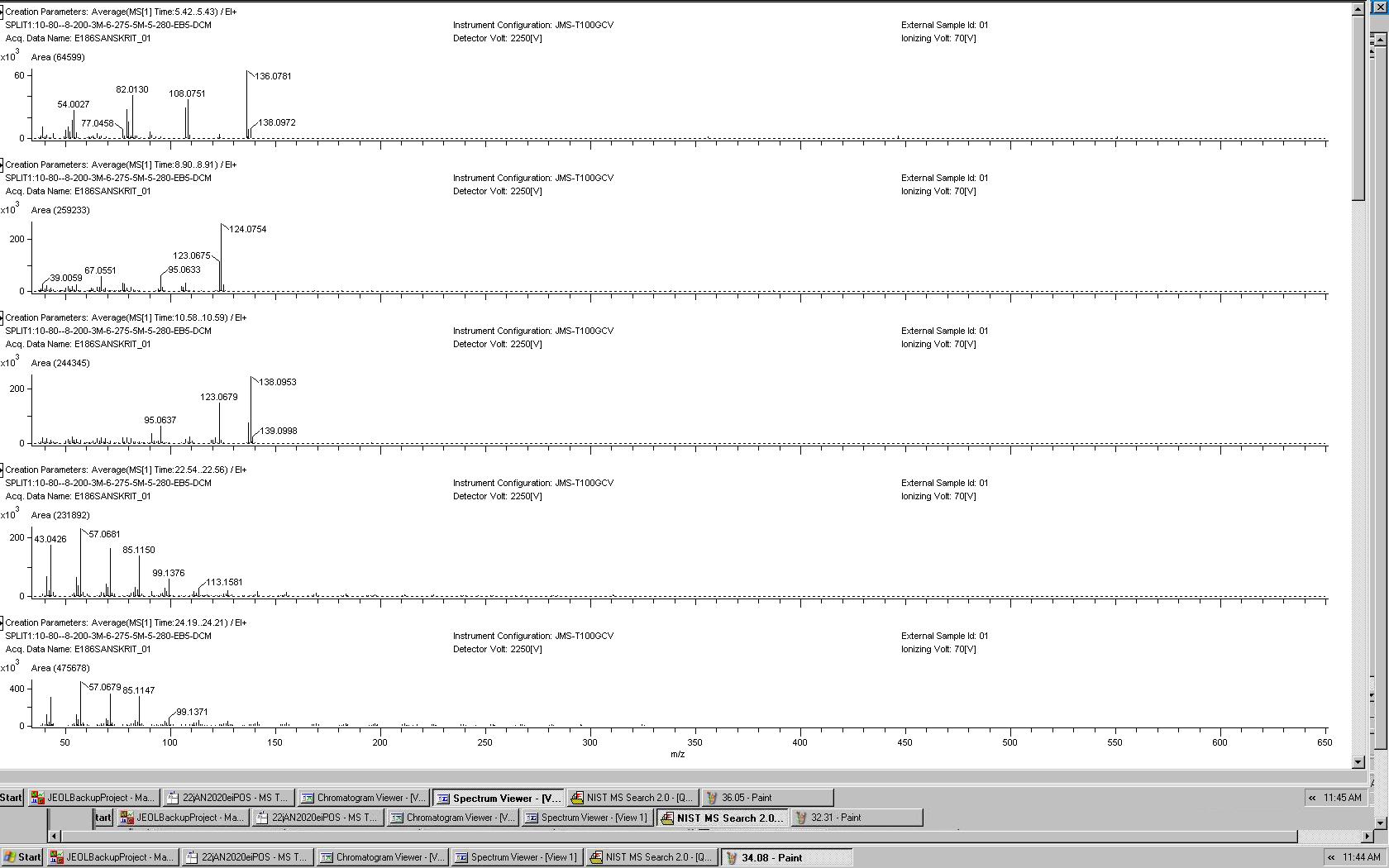

Supplement: Supplementary file 1 [file molecules-27-07476-s001.zip › molecules-1923168-supplementary/GC MS data/MS1.JPG]

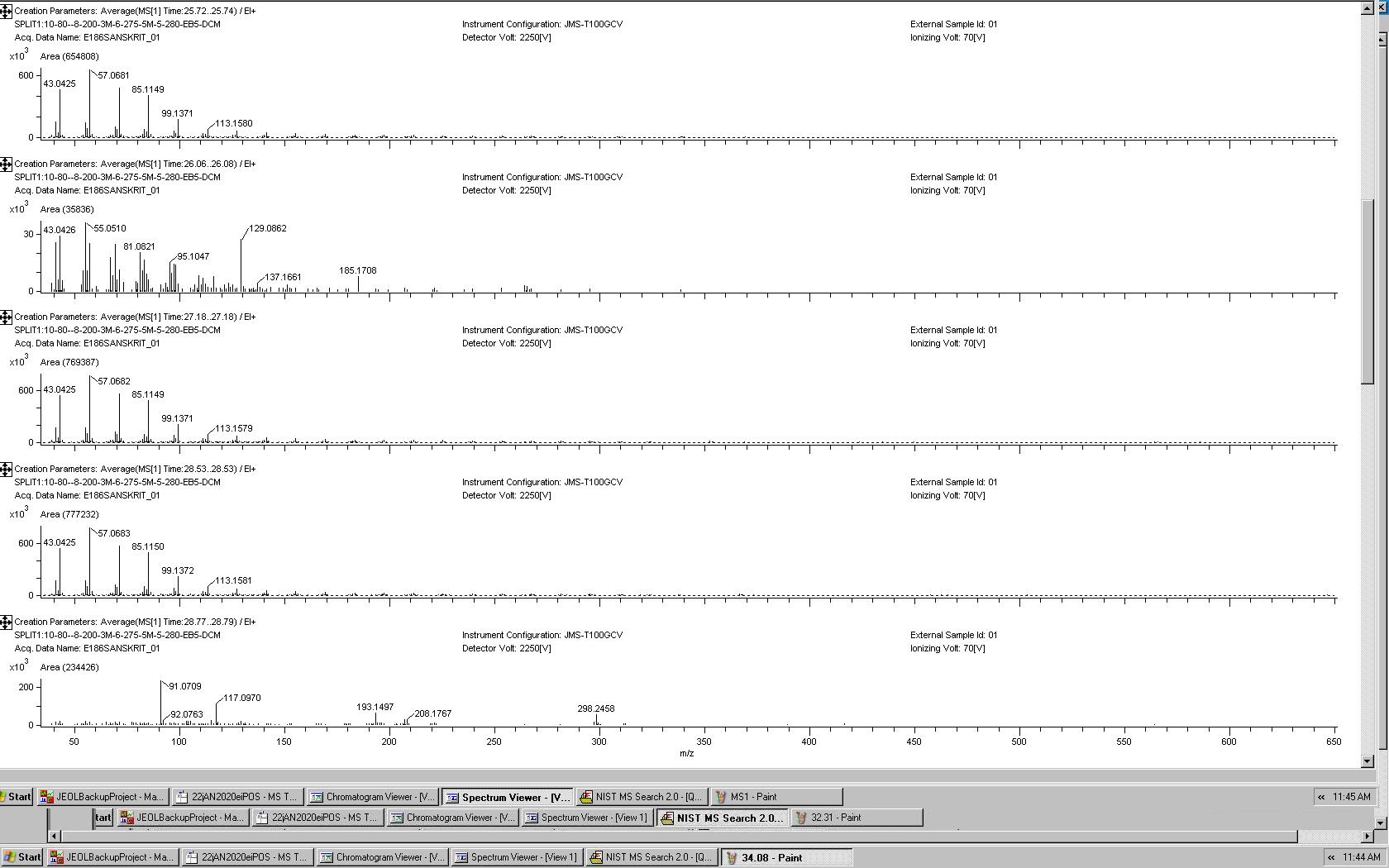

Supplement: Supplementary file 1 [file molecules-27-07476-s001.zip › molecules-1923168-supplementary/GC MS data/MS2.JPG]

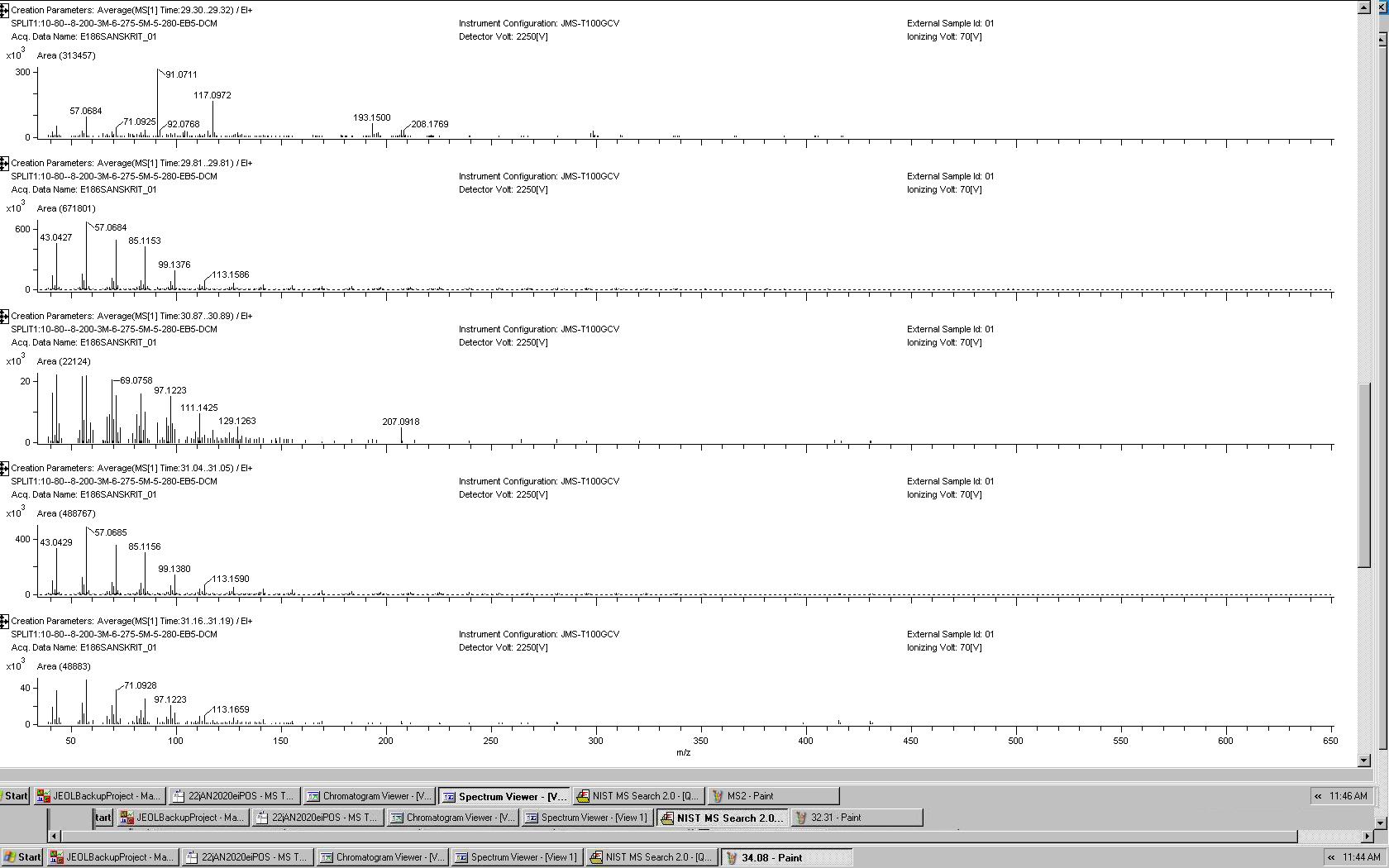

Supplement: Supplementary file 1 [file molecules-27-07476-s001.zip › molecules-1923168-supplementary/GC MS data/MS3.JPG]

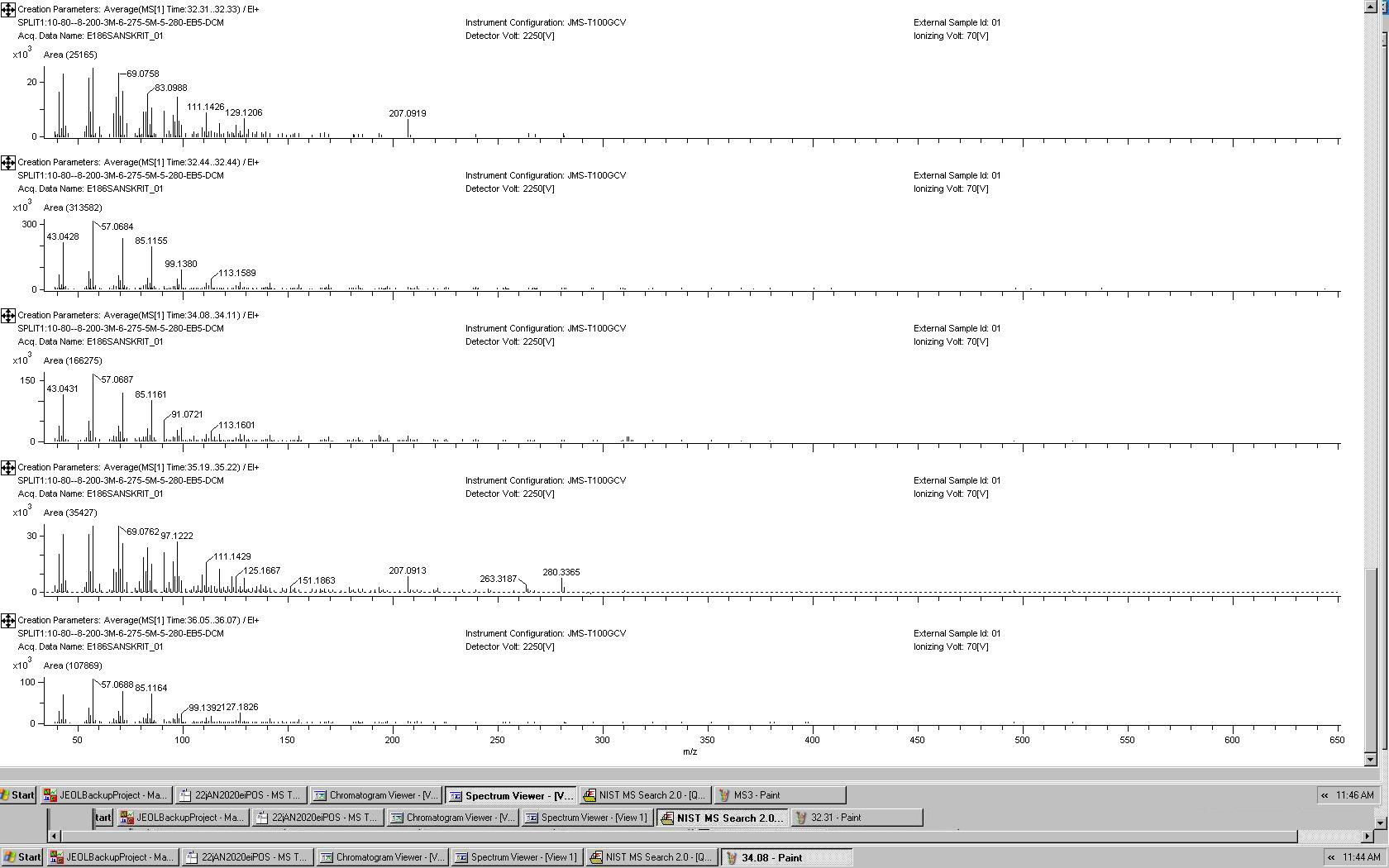

Supplement: Supplementary file 1 [file molecules-27-07476-s001.zip › molecules-1923168-supplementary/GC MS data/MS4.JPG]

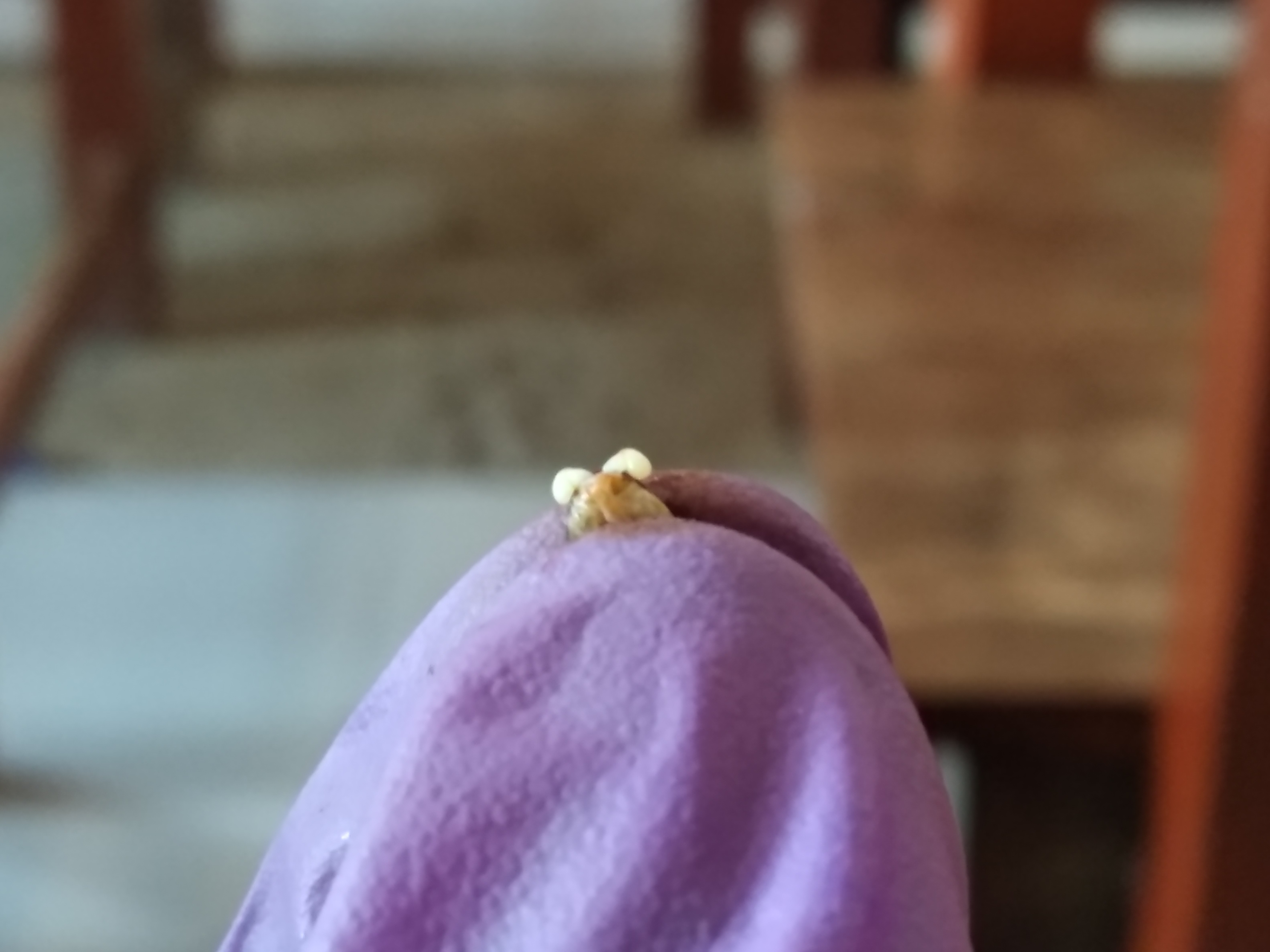

Supplement: Supplementary file 1 [file molecules-27-07476-s001.zip › molecules-1923168-supplementary/IMG_20190322_140632_HHT.jpg]

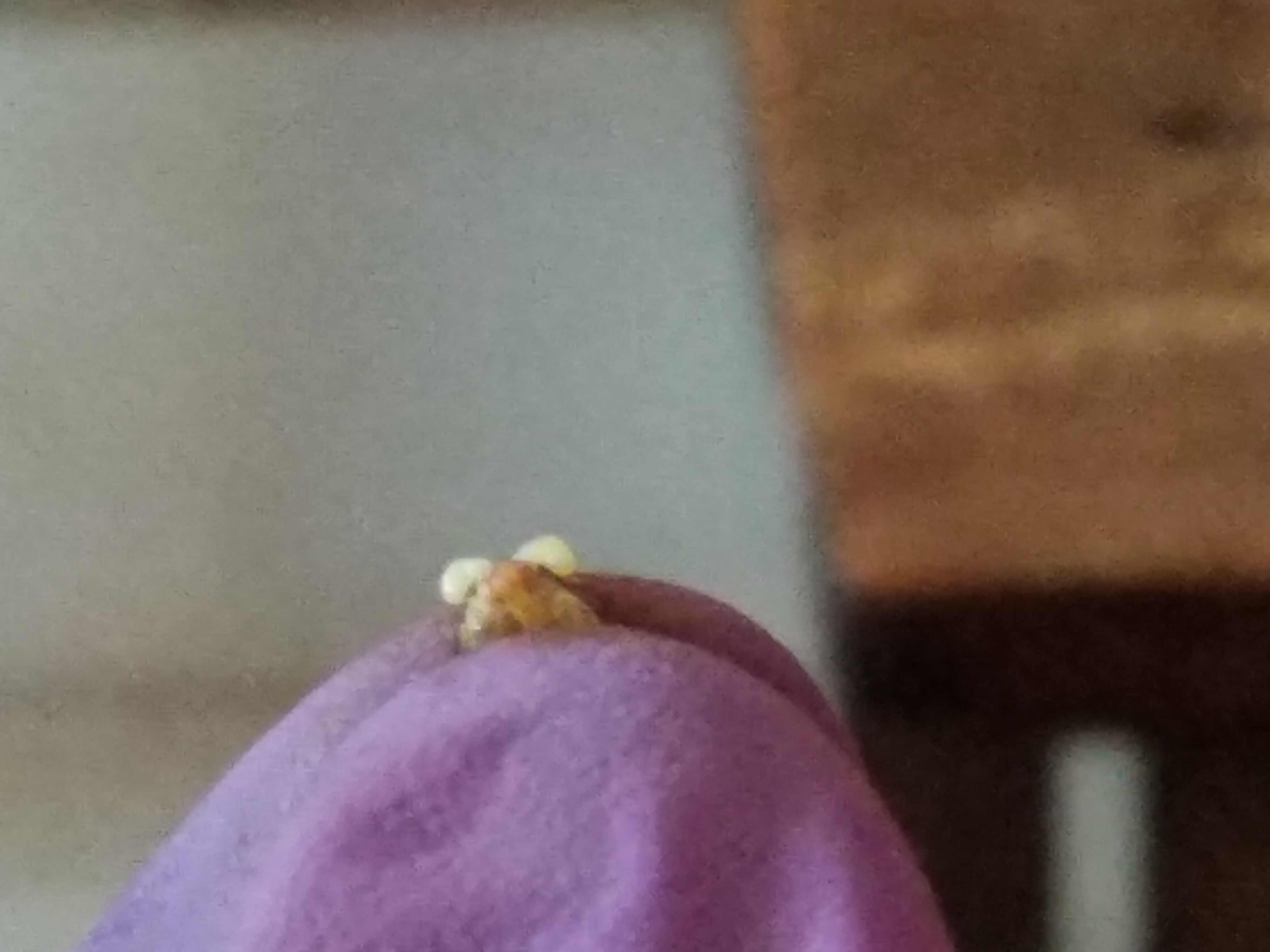

Supplement: Supplementary file 1 [file molecules-27-07476-s001.zip › molecules-1923168-supplementary/IMG_20190322_140650.jpg]
